# Altered network and rescue of human neurons derived from individuals with early-onset genetic epilepsy

**DOI:** 10.1038/s41380-021-01104-2

**Published:** 2021-04-22

**Authors:** Priscilla D. Negraes, Cleber A. Trujillo, Nam-Kyung Yu, Wei Wu, Hang Yao, Nicholas Liang, Jonathan D. Lautz, Ellius Kwok, Daniel McClatchy, Jolene Diedrich, Salvador Martinez de Bartolome, Justin Truong, Ryan Szeto, Timothy Tran, Roberto H. Herai, Stephen E. P. Smith, Gabriel G. Haddad, John R. Yates, Alysson R. Muotri

**Affiliations:** 1 Department of Pediatrics, University of California San Diego, La Jolla, CA USA; 2 Department of Chemical Physiology, The Scripps Research Institute, La Jolla, CA USA; 3 Center for Integrative Brain Research, Seattle Children’s Research Institute, Seattle, WA USA; 4 Graduate Program in Neuroscience, University of Washington, Seattle, WA USA; 5 Experimental Multiuser Laboratory, Graduate Program in Health Sciences, School of Medicine, Pontifícia Universidade Católica do Paraná, Curitiba, Paraná Brazil; 6 Department of Neurosciences, University of California San Diego, La Jolla, CA USA; 7 Kavli Institute for Brain and Mind, University of California San Diego, La Jolla, CA USA; 8 Center for Academic Research and Training in Anthropogeny (CARTA), La Jolla, CA USA

**Keywords:** Stem cells, Neuroscience, Drug discovery, Psychiatric disorders

## Abstract

Early-onset epileptic encephalopathies are severe disorders often associated with specific genetic mutations. In this context, the CDKL5 deficiency disorder (CDD) is a neurodevelopmental condition characterized by early-onset seizures, intellectual delay, and motor dysfunction. Although crucial for proper brain development, the precise targets of CDKL5 and its relation to patients’ symptoms are still unknown. Here, induced pluripotent stem cells derived from individuals deficient in CDKL5 protein were used to generate neural cells. Proteomic and phosphoproteomic approaches revealed disruption of several pathways, including microtubule-based processes and cytoskeleton organization. While CDD-derived neural progenitor cells have proliferation defects, neurons showed morphological alterations and compromised glutamatergic synaptogenesis. Moreover, the electrical activity of CDD cortical neurons revealed hyperexcitability during development, leading to an overly synchronized network. Many parameters of this hyperactive network were rescued by lead compounds selected from a human high-throughput drug screening platform. Our results enlighten cellular, molecular, and neural network mechanisms of genetic epilepsy that could ultimately promote novel therapeutic opportunities for patients.

## Introduction

Epilepsy is a common neurological disorder in early childhood, leading to a high burden of cognitive and behavioral comorbidity [1]. Seizures reflect a transient, abnormal, and synchronous hyperactivity of a neuronal population due to an imbalance between inhibitory and excitatory neurotransmission, resulting in tonic depolarizations or rhythmic burst discharges [2, 3]. Therefore, epilepsy is not a singular disease entity, but a multifactor and symptomatologically diversified condition that may result from different causes ranging from inherited mutations to structural brain abnormalities [4]. In this context, single-gene epilepsies are mainly composed of mutations in the *PRRT2, SCN1A, KCNQ2, SLC2A1*, or *CDKL5* genes [5].

The cyclin-dependent kinase-like 5 (CDKL5) gene localizes on chromosome Xp22 and encodes for a serine/threonine kinase highly expressed in the central nervous system [6–10]. Mutations in this gene cause CDKL5 deficiency disorder (CDD), characterized by neurodevelopmental delay, motor dysfunction, autistic features, and early-onset intractable seizures, a defining trait that led to the standalone classification of this pathology [11–19]. Due to *CDKL5*’s X-linkage, female heterozygous CDD patients show a spectrum of phenotypes based on the mosaicism generated by X-chromosome random inactivation. Hemizygous males, with a nonfunctional copy of the gene, likely display more severe clinical symptoms [20]. Nonetheless, both male and female CDD patients exhibit various mutations (translocations, nonsense, missense, frameshift, and splice variants), mostly found in the N-terminal kinase domain, resulting in an absent or malfunctioning CDKL5 protein [21–28].

CDKL5 is crucial for proper brain development and neuronal function [15, 29–31], but its precise targets in relevant cell types are still yet to be determined. To clarify the effects of CDKL5 loss-of-function and the etiology of CDD, several mouse models have been generated [32–34]. Specific behavioral abnormalities such as cognitive deficits and impaired motor control were described. However, none of the rodent models showed a consistent and robust recapitulation of the human condition, including the absence of spontaneous seizures in early development [35]. These phenotypes were accompanied by neuroanatomical variations, such as altered dendritic arborization, spine defects, and compromised neuronal connectivity, along with disruption of signaling pathways [30–33, 36–42].

Despite the overall research efforts, little is known about the molecular basis of seizures in human brain development, leading to an unsatisfactory clinical translation and no effective treatment [43]. Here, we used a human stem cell-based model to elucidate the molecular, cellular, and network mechanisms of CDD neuropathology. Proteomic and phosphoproteomic characterization of induced pluripotent stem cell (iPSC)-derived neural cells and cortical organoids revealed dysregulation of pathways involved in neural development, cytoskeleton-related proteins and connectivity. In line with the molecular alterations, we observed consistent proliferation and viability phenotypes in neural progenitor cells (NPCs), and alterations in the morphology and synaptogenesis of cortical excitatory neurons. The dynamic recording of CDD neural networks in cortical organoids revealed early hyperexcitation caused by intrinsic electrophysiological properties of glutamatergic neurons, and an overly synchronized network. Finally, these findings guided us to develop a human high-throughput (HT) drug screening platform to identify novel candidate drugs able to rescue the altered functional network in this early-onset genetic epilepsy model that could be used for other types of epileptic syndromes.

## Results

### Proliferation defects and increased cell death in CDD neural progenitors

**Fig. 1:**
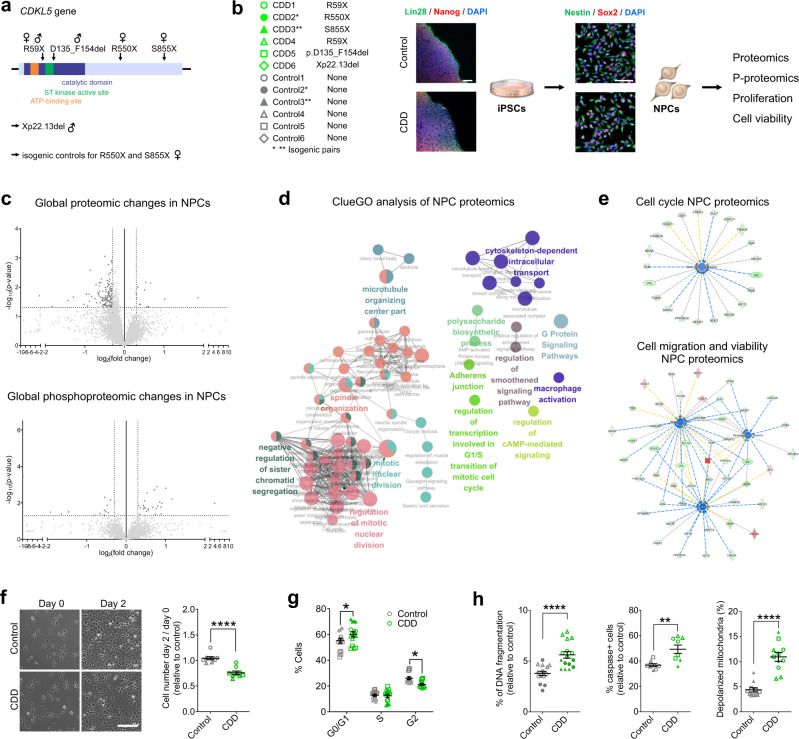
Altered proliferation and viability in CDD progenitor cells. **a** Schematic representation of the *CDKL5* gene showing the mutations exhibited by CDD cell lines used in the study. **b** Left, each cell line is represented by an individual colored symbol that is used throughout the study. CDD and related controls share the same symbol; filled symbols represent isogenic cells. Right, overview of the assays performed using NPCs. Representative images showing stage-specific protein expression in iPSCs and NPCs. Scale bar, 100 µm. **c** Volcano plot illustrating the global proteomic (top) and phosphoproteomic (bottom) changes in CDD vs. control NPCs. The *y*-axis shows the −log10 of the *t*-test *p* value. A *p* value of 0.05 and a|log2(fold change)| of 0.5 are indicated by dotted lines (Control, *n* = 4 cell lines; CDD, *n* = 4 cell lines; each cell line was derived from a different subject). **d** ClueGO analysis of differentially expressed proteins in CDD NPCs. The size of the circles indicates its significance, and similar terms are grouped with the same color (Control, *n* = 4 cell lines; CDD, *n* = 4 cell lines). **e** Ingenuity pathway analysis (IPA) showing differentially expressed proteins (DEPs) in the NPC dataset and the predicted effect of these changes. Top, DEPs involved in cell-cycle progression. Bottom, predicted effects on development of neurons, and cell viability. Blue means predicted inhibition, green indicates observed downregulation, and red indicates observed upregulation (Control, *n* = 4 cell lines; CDD, *n* = 4 cell lines). **f** Representative phase-contrast images showing the differences in confluency between CDD and control NPCs at day 2 and, ratio of CDD NPC number at day 2 over day 0 relative to control. Scale bar, 100 μm (Control, *n* = 9, 3 cell lines; CDD, *n* = 9, 3 cell lines; minimum of two technical replicates per cell line; two-tailed Mann–Whitney *U* test, ****p < 0.0001). **g** Percentage of cells at different phases of the cell cycle compared to control (Control, *n* = 16, 4 cell lines; CDD, *n* = 16, 4 cell lines; four technical replicates per cell line; two-way ANOVA, *p < 0.05). **h** Cell death analysis of CDD NPCs compared to control (Control, *n* = 16, 8, and 11, respectively, for fragmented DNA, caspase+ cells and depolarized mitochondria, 4 cell lines; CDD, *n* = 16, 8, and 11, respectively, for fragmented DNA, caspase+ cells and depolarized mitochondria, 4 cell lines; minimum of two technical replicates per cell line; two-tailed Mann–Whitney *U* test, **p = 0.0110, ****p < 0.0001). In all assays, each cell line was derived from a different subject. In **f–h**, data are shown as mean ± s.e.m.; individual values are indicated by dots where each symbol represents a subject.

The impact of CDKL5 deficiency on human neurodevelopment was investigated after cellular reprogramming. Skin fibroblasts derived from six CDD patients, carrying five distinct *CDKL5* mutations, were reprogrammed into iPSCs and fully characterized (Figs. 1a and  S1). All CDD iPSC lines do not express a functional CDKL5 protein (Figs. S1 and S2a–c). This cohort includes additional iPSCs from sex-matched related controls, along with isogenic control clones for female subjects based on random X-chromosome inactivation during cellular reprogramming (Fig. S1f, g) [44]. Each cell line is depicted with a color-coded symbol for easy visualization of the data on individual genotypes. A description of all experiments performed and cell lines used can be found in Table S1.

The iPSCs were differentiated into NPCs. Considering the CDKL5 kinase function, we first performed mass spectrometry-based quantitative proteomics and phosphoproteomics to gain insights into its targets in a relevant cell type (Fig. 1b–e). We identified and quantified 6505 proteins and determined differentially expressed protein (DEP) as proteins with *t*-test *p* value < 0.05 and log2(fold change) cutoff of >0.3 (12 proteins) or <−0.3 (124 proteins) (Tables S2 and S3). Although we have not observed a significant enrichment of gene ontology (GO) terms in the phosphoproteomics dataset, proteomic (non-phospho) analysis of CDD NPCs showed enrichment of microtubule-based process (23 proteins) among the downregulated proteins (FDR *q* value for overlap: 4.14E − 4, 23 proteins). Many of these proteins (TUBGCP5, HAUS3, NDE1, NDC80, FBXO5, CUL7, ID2, KIF15, CHECK2) are also related to the mitotic cell cycle. Dysregulated phosphorylation or differential expression of proteins regulating cellular structures might hamper the proper neuronal development and plasticity. The DEP changes also pointed to an impairment in cell viability and neuronal development in CDD (Fig. 1d, e). Therefore, the proteomic signature pathways found in NPCs encouraged us to further evaluate the proliferative aspect of these cells. By assessing in vitro cell number over time, we observed that CDD NPCs proliferate slowly compared to controls. Similar results of an altered cell cycle were obtained using image-based approaches (Fig. 1f, g). In addition, increased DNA fragmentation, mitochondrial depolarization, and caspase activity were observed (Fig. 1h). Together with an unaltered proportion of neurons following differentiation compared to control (Fig. S2d), these results suggest compromised proliferation and increased cell death in CDD neural cultures.

### Phosphoproteomics analysis predicts alterations in the development and mTOR activation in CDD neurons

**Fig. 2:**
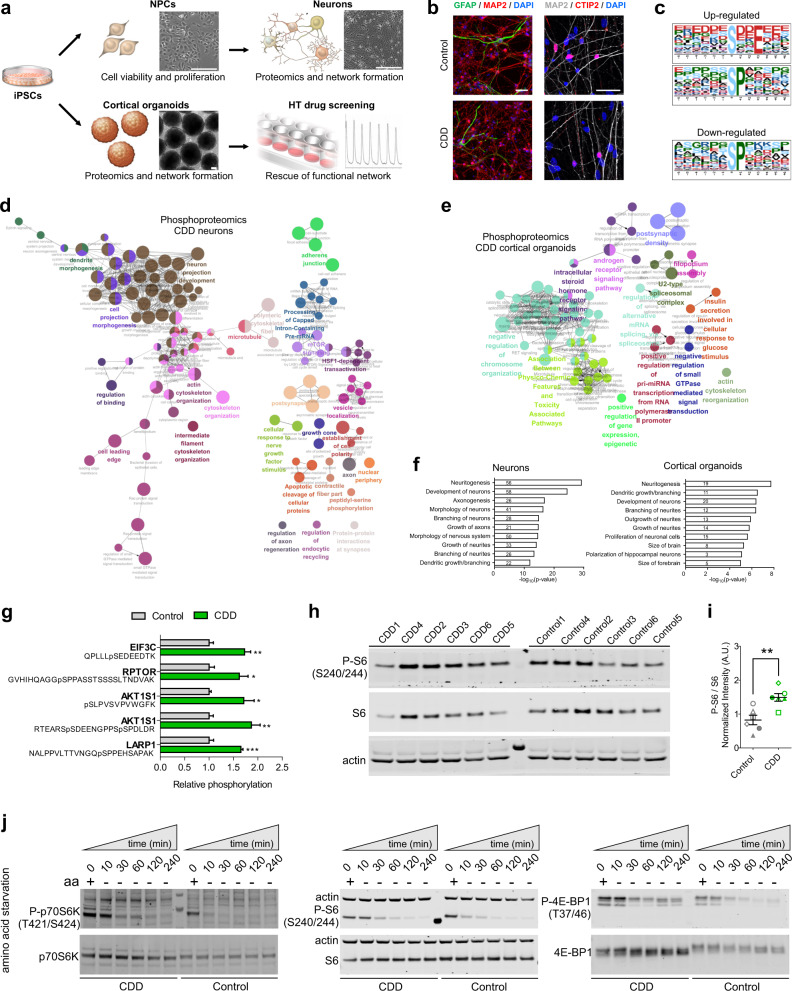
Proteomics and phosphoproteomics analysis reveal mTOR alterations in CDD neural cells. **a** Schematic diagram showing the iPSC differentiation into neural lineages, and summarizing the assays performed with each cell type. Representative phase-contrast images of NPCs, neurons, and cortical organoids are shown. Scale bar, 100 µm. **b** Stage-specific protein expression in 6-week-old cortical neural cultures. Scale bar, 100 µm. **c** Motif analysis of up- and downregulated phosphopeptides in CDD 6-week-old neurons (Control, *n* = 5 cell lines; CDD, *n* = 5 cell lines). **d** ClueGO analysis of differentially expressed phosphoproteins in 6-week-old CDD neurons (Control, *n* = 5 cell lines; CDD, *n* = 5 cell lines). **e** ClueGO analysis of differentially expressed phosphoproteins in 2-month-old CDD cortical organoids (Control, *n* = 4 cell lines; CDD, *n* = 4 cell lines). The size of the circles indicates its significance, and similar terms are grouped as the same color. **f** Top enriched terms for significantly regulated phosphoproteins in the category of nervous system development and function in IPA. **g** Relative phosphorylation of proteins at downstream of mTOR signaling pathway in 6-week-old neurons. Relative ratios of phosphopeptide intensity normalized to non-phosphoprotein intensity are shown (Control, *n* = 5 cell lines; CDD, *n* = 5 cell lines; unpaired *t*-test, each row was analyzed individually without assuming a consistent SD, *p < 0.05, **p < 0.01, ***p < 0.001). **h**, **i** Quantification of phosphorylated-S6/total S6 ratio from Western blot using protein lysates from 6-week-old CDD and control neurons (Control, *n* = 6 cell lines; CDD, *n* = 6 cell lines; two-tailed Mann–Whitney *U* test, **p = 0.0087). The band intensity was quantified using a LI-COR imaging system and the P-S6 to S6 ratio was calculated for each sample. **j** Time-dependent responses of CDD and control cells upon amino acid (aa) starvation. CDD and control NPCs were incubated with media containing or lacking amino acids for the indicated times. mTORC1 activity was determined by Western blot with antibodies against phospho-p70S6K, phospho-S6, phospho-4E-BP1 and total p70S6K, S6 and 4E-BP1 (Control, *n* = 3 cell lines; CDD, *n* = 3 cell lines). In **g** and **i**, data are shown as mean ± s.e.m. Individual values are indicated by dots where each symbol represents a subject. CDD and related controls share the same symbol and filled symbols represent isogenic cells.

To further understand the impact of CDKL5 absence in the brain, we differentiated the cells into cortical neurons and cortical organoids [45–48] (Fig. 2a, b). The CDKL5 protein was not detected in iPSCs, mildly expressed in NPCs, and upregulated in neurons, as previously described in mouse [10, 49] (Fig. S2a–c). The samples were analyzed by mass spectrometry following the phosphopeptide enrichment. In 2D 6-week-old neurons, we identified and quantified 4115 phosphopeptides from 1485 proteins. After normalization of the phosphopeptide intensity, we determined the most significantly changed phosphopeptides as those with |log2(fold change)| > 0.5 and *t*-test *p* value < 0.05 (Tables S2 and S3). In 2-month-old cortical organoids, we identified and quantified 6769 phosphopeptides from 2212 proteins (|log2(fold change)| > 0.3 and *t*-test *p* value < 0.05, 120 phosphopeptides from 158 proteins, FDR ≤ 0.05) (Tables S2 and S3).

Motif analyses detected significantly enriched sequences around the phosphosites, with over-representation of pSXXE among the upregulated phosphopeptides (Fig. 2c), which is phosphorylated by acidophilic kinases such as CK2. Ingenuity pathway analysis and kinase enrichment analysis revealed that phosphoproteins known to be phosphorylated by CK2 were increased in CDD neurons, suggesting that CK2 was over-activated. Over-representation of pSP motif was also found among the downregulated phosphopeptides in CDD neurons (Fig. 2c). CDKL5 was not detected as an upstream kinase regulator, presumably due to the limited knowledge about its substrates. Although downregulation of phosphoproteins might be linked with inhibition of other proline-directed kinases, a subset of the downregulated phosphoproteins may be the direct substrates of CDKL5.

The phosphoproteomic analysis revealed several dysregulated phosphoproteins in CDD neurons and organoids (Figs. 2d–g and S2e–k). Altered phosphorylation of microtubule-associated proteins such as MAP1A, MAP1B, MAP2, and collapsin response mediator protein family proteins (DPYSL2 and DPYSL3) was also found in these cell types; along with cytoskeleton organization proteins, such as CTTN. The overall enriched terms in neurons and organoids include neuritogenesis, morphology, migration, synaptogenesis, and neurotransmission (Figs. 2f,  S2f and Tables S4–S6).

Among the downregulated proteins observed in the proteomic analysis, the gamma-aminobutyric acid signaling pathway was also over-represented (Fig. S2i). For the upregulated proteins, markedly enriched GO terms were lysosome, glycosphingolipid metabolic process, and aminoglycan catabolic process (Tables S4–S6). Upregulated proteins such as GALNS, GM2A, EPHA2, RHOG, HEXA, and HEXB are involved in the degradation of chondroitin sulfate, a sulfated glycosaminoglycan that participates in neurite outgrowth. Moreover, in the phosphoproteomic analyses of cortical neurons, we found enrichment of mTOR-pathway-related phosphoproteins (Figs. 2g and S2j). Increased phosphorylation of proteins such as AKT1S1 (also known as PRAS40), EIF3C, RPTOR, and LARP1 suggested that the pathway is activated in CDD cells. Thus, we independently investigated and confirmed the increased phosphorylation of ribosomal subunit S6 protein levels in CDD neurons (Figs. 2h, i and  S2k).

To further validate these results, we tested whether CDD neural cells are impaired in their ability to turn off mTORC1 downstream proteins upon amino acid starvation [50]. When cultured with medium lacking amino acids, control cells shut off mTORC1 activity faster and to lower levels than CDD cells. In contrast, CDD cells are significantly impaired in their response to amino acid removal, retaining mTORC1 activity levels for longer. Unlike control cells, in which the phosphorylation levels of p70S6K, S6, and 4E-BP1 are almost inexistent after 60 min of amino acid withdrawal, CDD cells have a delayed response exhibiting only a partial drop (Figs. 2j and  S2l). Therefore, in addition to alterations in cytoskeleton organization and neurotransmission, the mTOR-pathway activation predicted by proteomic and phosphoproteomic signatures of CDD neurons was confirmed by amino acid starvation in CDD cells.

### CDD neurons display morphological abnormalities and impaired migration

**Fig. 3:**
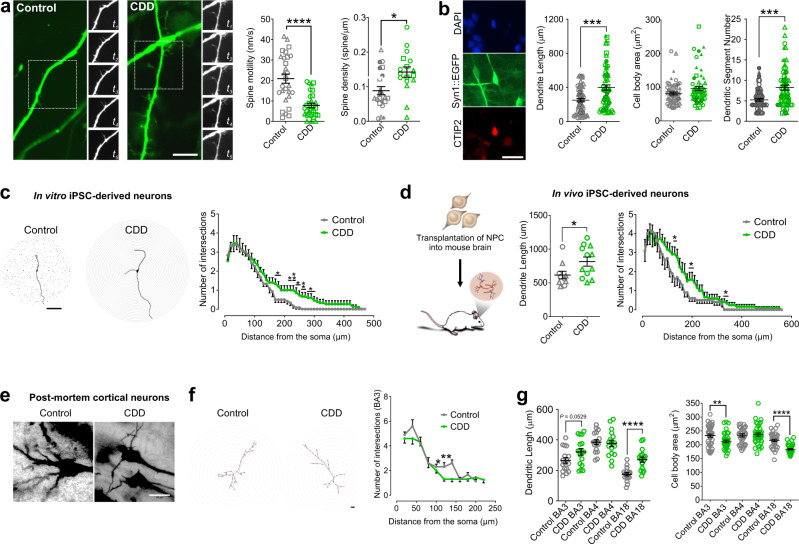
Cellular and morphological defects in CDD neurons. **a** Representative images of neuronal spine-like structures in 8-week-old CDD and control neurons transduced with Synapsin1 promoter-driven EGFP expression. The time series images were taken every 5 min. Scale bar, 10 μm (Motility: Control, *n* = 28, 3 cell lines; CDD, *n* = 30, 3 cell lines; Density: Control, *n* = 22, 5 cell lines; CDD, *n* = 17, 3 cell lines; minimum of four technical replicates per cell line; two-tailed Mann–Whitney *U* test, *p < 0.05, ****p < 0.0001). **b** Left, representative immunostaining images of EGFP+ and CTIP2+ neurons used for neuronal tracing. CTIP2 was used as cortical layers V/VI marker (red). Right, morphometric analyses showing significant differences in total dendritic length and dendritic segment number, but not in cell body area of 8-week-old CDD neurons compared to controls. Scale bar, 20 μm (Control, *n* = 64, 5 cell lines; CDD, *n* = 64, 5 cell lines; minimum of three technical replicates per cell line; two-tailed Mann–Whitney *U* test, ***p = 0.0004 for dendritic length and, ***p = 0.0002 for dendritic segment number). **c** Representative images of tracings from CDD and control neurons in vitro. Graph showing Sholl analysis of the dendritic complexity of EGFP+ neurons. Number of intersections refers to the number of dendrites intersecting concentric circles spaced 10 μm apart starting from the cell body (Control, *n* = 12, 5 cell lines; CDD, *n* = 15, 5 cell lines; unpaired *t*-test, each row was analyzed individually without assuming a consistent SD, *p < 0.05, **p < 0.01). **d** Left, schematic of the chimeric human-mouse brain approach. Middle, morphometric analysis showing significant difference in total dendritic length between CDD and control neurons at 6 months after transplantation. Right, Sholl analysis of the dendritic complexity of EGFP+ human neurons in vivo (Control, *n* = 10, 2 cell lines; CDD, *n* = 12, 2 cell lines; dendritic length: two-tailed Mann–Whitney *U* test, *p < 0.0358; Sholl analysis: unpaired *t*-test, each row was analyzed individually without assuming a consistent SD, *p < 0.05). **e** Representative images of postmortem cortical layers V/VI pyramidal neurons using Golgi staining. Scale bar, 25 μm. **f** Neuronal tracing of postmortem neurons from CDD and control. The tissue was collected at 5- and 6-year-old, respectively, for CDD and control subjects. Left, representative images of tracings. Right, Sholl analysis of the dendritic complexity of silver-stained neurons. Number of intersections refers to the number of dendrites intersecting concentric circles spaced 20 μm apart starting from the cell body. Scale bar, 20 μm (Control, *n* = 10 neurons, 1 subject; CDD, *n* = 10 neurons, 1 subject; unpaired *t*-test, *p < 0.05, **p < 0.01). **g** Morphometric analyses of postmortem cortical neurons showing differences in total dendritic length and cell body area between CDD and control (Dendritic length: Control, *n* = 17 neurons, 1 subject; CDD, *n* = 17 neurons, 1 subject; cell body area: Control, *n* = 40 neurons, 1 subject; CDD, *n* = 40 neurons, 1 subject; two-tailed Mann–Whitney *U* test, **p < 0.01, ****p < 0.0001). Data are shown as mean ± s.e.m. Individual values are indicated by dots where each symbol represents a subject. CDD and related controls share the same symbol and filled symbols represent isogenic cells.

Cytoskeletal proteins structurally support the neuronal dendrites and axons, and play essential roles in neuronal morphogenesis, plasticity, and migration. Since cytoskeletal protein alterations were highlighted by the proteomics, we initially measured neurite outgrowth and spine-like dynamics. Neurons stably transfected to express EGFP by Synapsin1 promoter self-inactivating lentivirus were dissociated, re-platted, and had their neurites recorded over time. We noticed that the spine-like motility was significantly reduced, while spine-like density was increased in CDD neurons (Fig. 3a). These cellular observations agreed with the alterations detected on microtubule dynamics and cytoskeleton organization.

The total dendritic length and complexity of CDD neurons were significantly higher than controls (Figs. 3b, c and  S3a). To determine whether these phenotypes were cell-autonomous, we transplanted human NPCs, previously transduced with SYN1:EGFP to label neurons, into newborn mouse brains (see “Methods” for details). Since the brain environment promotes a faster neuronal maturation of human transplanted cells [51, 52], we used this strategy to highlight the cell-autonomous phenotypes of CDD neurons in a more pronounced fashion for extended periods (Figs. 3d and  S3b). Ten to twenty newborn mice were transplanted, and a fraction of the human cells integrates individually or in small clusters into the host brain with similar morphometric dimensions as adjacent host cells. Six months after grafting, the transplanted cells were integrated and differentiated in the mouse brain. The EGFP+ human neurons were 3D-reconstructed, and the morphological features were analyzed. A significant increase in dendritic length was found in transplanted CDD neurons, in agreement with the in vitro CDD morphological alterations (Figs. 3d and S3c).

In an attempt to place our findings in the broader context of the cortical morphology in human participants at the cellular level, we conducted orthogonal validation experiments using postmortem brain tissues from gender-, age-, and hemisphere-matched CDD and control subjects (Figs. 3e–g and S3d–f). Considering the limited availability of samples, the human tissue was used to test predictions based on the neuronal differences found in vitro, validating possible alterations in the morphology of cortical neurons. We observed heterogeneity of results in vivo depending on the specific cortical area under investigation (Broadman area, BA) and, most importantly, on the individual’s age. Similar to our in vitro data, BA3 and BA18 cortical neurons in postmortem tissue from a young CDD patient (5-year-old) displayed larger total dendritic length (Fig. 3g). However, these differences change in neurons obtained from an older patient (30-year-old. Fig. S3f), indicating that the development stage in which CDD neuronal morphology is assessed is crucial for comparisons. In addition, we also observed that CDD proliferation and migration alterations lead to smaller cortical organoids and migration defects compared to controls (Fig. S3g, h).

### CDKL5 deficiency impacts synaptogenesis and promotes early network hyperexcitability

**Fig. 4:**
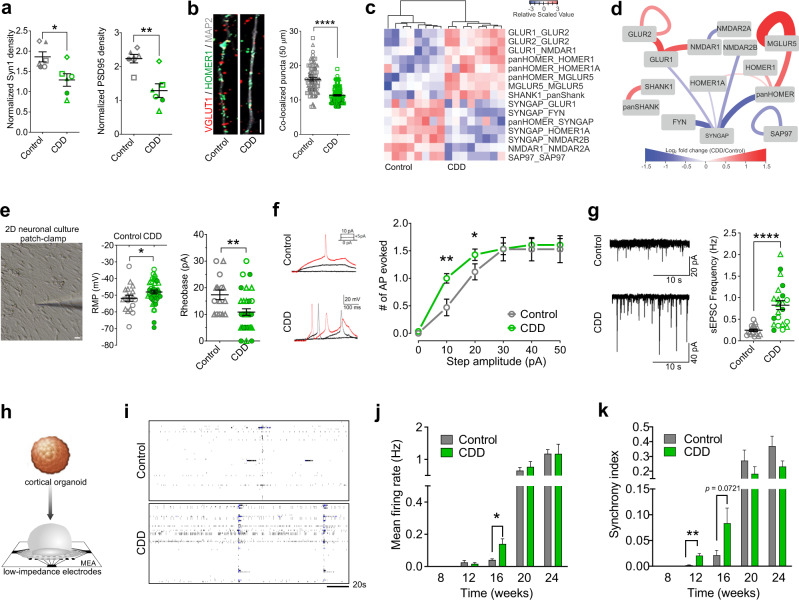
Altered synaptic and network activity in CDD neurons. **a** Expression levels of pre- and post-synaptic markers (respectively, Synapsin1 and PSD95) in 8-week-old CDD neurons compared to control, assessed by Western blot analysis (Control, *n* = 6 cell lines; CDD, *n* = 6 cell lines; two-tailed Mann–Whitney *U* test, *p = 0.0260, **p = 0.0043). **b** Representative images (left) and puncta quantification (right) of MAP2+ neurons stained for pre- and post-synaptic markers (VGLUT1 and HOMER1, respectively) (Control, *n* = 83, 5 cell lines; CDD, *n* = 108, 5 cell lines; minimum of 13 technical replicates per cell line, two independent experiments; two-tailed Mann–Whitney *U* test, ****p < 0.0001). Scale bar, 5 μm. **c** Heatmap of all interactions significantly different between CDD and control cortical organoids by ANC∩CNA. Rows indicate interactions, columns indicate experimental replicates. Data are clustered by column and scaled by row to represent intensity values that vary over three orders of magnitude (Control, *n* = 8, 2 cell lines; CDD, n = 8, 2 cell lines; four technical replicates per cell line, two independent experiments). **d** Node-edge representation of interactions listed in **c**. Edge color and thickness indicates the magnitude of the log2 fold change between CDD and control organoids. Red means higher in CDD (Control, *n* = 8, 2 cell lines; CDD, *n* = 8, 2 cell lines; four technical replicates per cell line, two independent experiments). **e** Whole-cell patch clamping of differentiated neurons spread in the dish. Left, representative image of patch-clamp neurons. Scale bar, 10 μm. Right, resting membrane potential (RMP) and rheobase currents in CDD neurons compared to control (RMP: Control, *n* = 19, 2 cell lines; CDD, *n* = 42, 3 cell lines; Rheobase: Control, *n* = 15, 2 cell lines; CDD, *n* = 35, 3 cell lines; minimum of six technical replicates per cell line; two-tailed Mann–Whitney *U* test, *p = 0.0386, **p = 0.0037). **f** Left, representative action potential (AP) firing traces evoked from −60 mV in CDD and control neurons. Right, number of evoked APs in response to current steps (0–50 pA) from resting membrane potential (Control, *n* = 17 neurons, 2 cell lines; CDD, *n* = 28 neurons, 3 cell lines; minimum of four technical replicates per cell line; unpaired *t*-test, each row was analyzed individually without assuming a consistent SD, *p < 0.05, **p < 0.01). **g** Left, representative recordings of spontaneous excitatory synaptic currents of CDD and control neurons voltage-clamped at −60 mV. Right, sEPSC frequency observed in CDD neurons compared to control (Control, *n* = 17, 2 cell lines; CDD, *n* = 22, 3 cell lines; minimum of five technical replicates per cell line; two-tailed Mann–Whitney *U* test, ****p < 0.0001). **h** Schematic of cortical organoid electrophysiological recording using a multielectrode array (MEA). **i** Representative raster plots of CDD and control cortical organoid neural network activity at 16 weeks of differentiation. Functional characterization of CDD cortical organoids showing changes in the mean firing rate (**j**) and synchrony index (**k**) over time, compared to control (Control, *n* = 6, 2 cell lines; CDD, *n* = 6, 2 cell lines; three technical replicates per cell line; unpaired *t*-test, *p = 0.0111, **p = 0.0030). Data are shown as mean ± s.e.m. Individual values are indicated by dots where each symbol represents a subject. CDD and related controls share the same symbol and filled symbols represent isogenic cells.

Next, we investigated how the cellular and molecular alterations found in CDD would impact neuronal functionality and network formation. Decreased expression of pre- and post-synaptic protein markers (Syn1 and PSD95, respectively) was observed by Western blot (Figs. 4a and S4a), and independently confirmed by the reduced density of co-localized VGLUT1 and HOMER1 synaptic puncta on MAP2-positive processes of CDD neurons (Fig. 4b).

To determine how CDKL5 deficiency might affect the dynamic protein co-associations of glutamatergic synapse-associated proteins, we used quantitative multiplex co-immunoprecipitation (QMI) to measure the abundance of protein co-associations among a set of 22 synaptic proteins [53, 54]. We analyzed the protein content from cortical organoids derived from two CDD and two related control individuals in duplicate, two separate times to quantify the effect of batch, genetic background, and genotype. Principal component analysis and hierarchical clustering revealed a main effect of CDKL5 deficiency, with apparent clustering of CDD compared to controls. Within these major groups, technical replicates differentiated in parallel clustered more closely than genetically identical lines, suggesting moderate batch effects (Fig. S4b). Nonetheless, 15 protein co-associations were significantly different between control and CDD carriers (Fig. 4c, d). The amount of immunoprecipitated mGluR5_probe mGluR5, and mGluR5_Homer was strongly upregulated, as was the amount of GluR1_GluR1 and GluR1_GluR2, indicating altered composition and scaffolding of synaptic glutamate receptors. We also detected lower levels of many interactions involving SynGAP, indicating reduced incorporation of this activity-dependent signaling protein into post-synaptic receptor complexes. These data demonstrate widespread changes in synaptic protein networks downstream of CDD neurons with the possible implication in the neural network formation.

To determine the impact of disrupted glutamatergic signaling on developing neuronal networks, we started at a single-cell level and performed a whole-cell patch-clamp recording of iPSC-derived 2D monolayer cortical neurons (10-week-old neurons). While the resting membrane potential was increased, a significantly lower rheobase current was observed in CDD neurons (Fig. 4e). Rheobase is a measure of membrane potential, indicating the minimal current amplitude that results in action potential (AP). No changes were observed in the membrane capacitance, AP amplitude, and width (Fig. S4c). The higher number of APs evoked by +10, and +20-pA current injections and a lower rheobase suggest higher neuronal excitability in CDD neurons compared to controls (Fig. 4f). In addition, the CDD neurons exhibited significant increases in Na^+^ current (*I*_Na_) and transient A-type current (*I*_KA_) densities, and a negative shift of channel activation compared with their corresponding controls, supporting the hyperexcitability profile (Fig. S4d). Corroborating these findings, increased sEPSC frequency and decreased amplitude were observed in CDD cortical neurons (Figs. 4g and  S4e). Together, we revealed the intrinsic hyperexcitability and dysfunction of voltage-gated Na^+^ and K^+^ channels that underlie the electrical hyperactivity in CDD cortical neurons.

To further evaluate the cortical organoid functionality at a mesoscopic level, we performed weekly extracellular recordings of spontaneous electrical activity using multielectrode arrays (MEA). Around four cortical organoids were plated per well in a MEA plate containing 64 microelectrodes with 30 μm of diameter spaced by 200 μm (Fig. 4h). The spikes were defined by the event unit waveforms standard structure with typical refractory periods [48]. For ~6 months, cortical organoids exhibited consistent increases in electrical activity, as parametrized by channel-wise firing rate, burst frequency, and synchrony. Furthermore, we observed a significant increase in spike frequency and an overly synchronized network at the early stages of development in CDD neuronal networks (Figs. 4i–k and  S4f). Altogether, these results highlight the contribution of a specific gene in the emergence of a hyperexcitable and overly synchronized network.

### HT drug screening and functional rescue of CDD neural network

**Fig. 5:**
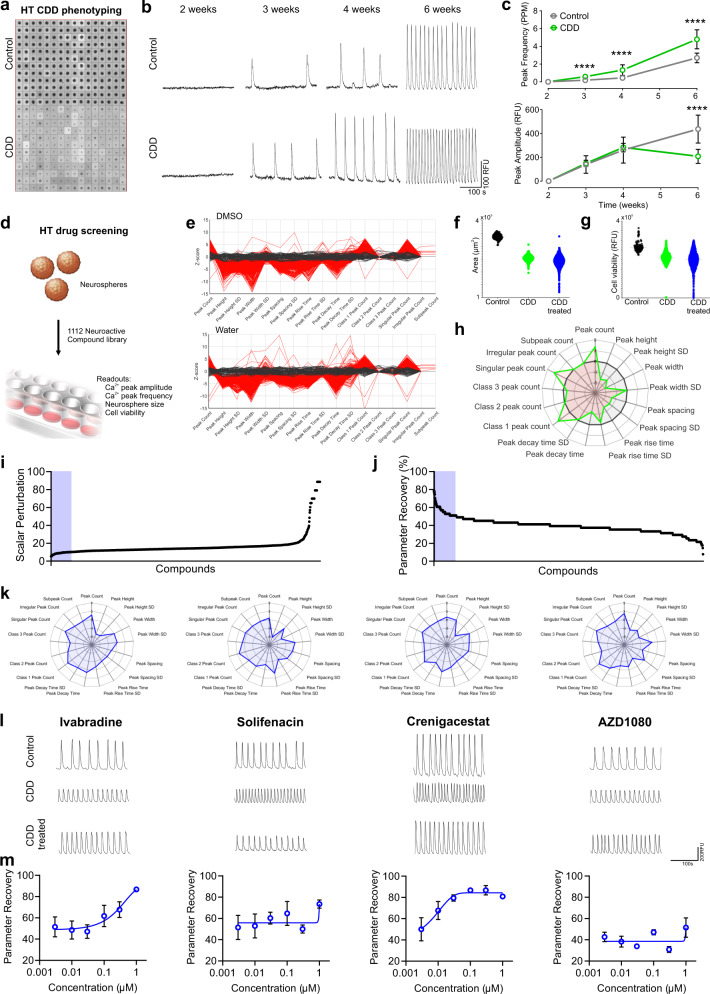
High-throughput screening and functional rescue of CDD neural network. **a** High-throughput (HT) format for CDD phenotypic screening in 384-well plates. Spheroids comprise a defined culture of neurons and astrocytes. **b** Functional characterization of CDD and control neural networks over time. Each peak represents a spontaneous calcium oscillation that correlates with synchronous neural activity. **c** Changes in peak amplitude and peak frequency in control and CDD cultures over time (2, 3, and 4 weeks *n* = 32 spheroids for control and CDD; 6 weeks *n* = 96 and 256 spheroids, respectively, for control and CDD; 1 cell line; ****p < 0.0001). Data are shown as mean ± s.d. **d** Schematic of the HT screening protocol. **e** *Z*-score of 17 calcium oscillation features in CDD neural networks (red) compared to control (black) at 6 weeks of differentiation. DMSO (0.1%, top) and water (bottom) were used as vehicle for the screening compounds. **f** Area of control, CDD-untreated and -treated spheroids after 6 weeks of differentiation. Chronic treatment with 1112 compounds was performed for three weeks (Control, *n* = 48, 1 cell line; CDD-untreated, *n* = 88, 1 cell line, CDD-treated, *n* = 1 spheroid per compound). Data are shown as mean ± s.d. **g** Cell viability of control (black), CDD-untreated (green) and CDD-treated (blue) spheroids after 6 weeks of differentiation. Chronic treatment with 1112 compounds was performed for 3 weeks (Control, *n* = 96, 1 cell line; CDD-untreated, *n* = 176, 1 cell line, CDD-treated, *n* = 2 spheroids per compound). Data are shown as mean ±;s.d. **h** Radar plot showing the functional calcium oscillation phenotype signature of CDD-untreated (green line) compared to control (black line) spheroids. Distribution of CDD-treated calcium activity according to the scalar perturbation (**i**) and recovery (**j**) parameters. In blue, top 50 compounds with lowest values for scalar perturbation and, top 25 compounds with more than 60% rescue for parameter recovery. **k** Radar plots showing the functional rescue of CDD calcium oscillation signature after treatment with selected compounds. **l** Representative calcium oscillation tracings from spheroids control, CDD-untreated and -treated with selected compounds. **m** Dose–response curve considering the parameter recovery percentage for selected compounds (*n* = 4 spheroids per concentration for each treatment, 1 cell line). Data are shown as mean ± s.e.m.

After identifying the hyperexcitability of CDD neural networks, we wondered if such phenotype could be pharmacologically reverted. For this purpose, we generated a HT drug screening platform composed of 3D neural spheroids in a 384-well format (one spheroid per well; Figs. 5 and S5). This platform was optimized to reduce variability within the genotypes, with spheroids displaying the same size across the plate, which is critical for a consistent and reproducible screening assay (Fig. 5a). Each spheroid contains a balanced population of cortical neurons and astrocytes expressing key markers of maturity and exhibits robust spontaneous, synchronous calcium oscillations [55]. Although both spheroid genotypes showed an increase in neural activity over time, the frequency of spontaneous calcium oscillations (number of peaks) was more pronounced in CDD compared to controls, starting at early stages of the development; while the amplitude (peak height) was decreased in CDD neurons (Figs. 5b, c and S5e). In this context, we strategically select calcium oscillations as a physiological readout to avoid disrupting the established 3D network [56], while evaluating the spheroid size and viability.

We screened a unique library of 1112 neuronal signaling compounds by chronically treating 3-week-old CDD spheroids in triplicate at 1 µM for three weeks (Figs. 5d, S5 and Table S7). Therefore, we determined the calcium peaks’ baseline features solely in the presence of each vehicle (DMSO or water) to avoid any confounding effect on the oscillatory activity (Figs. 5e and  S5a–d). Similarly, the result from each compound was normalized against its vehicle control (DMSO or water). To detect potential toxic effects of specific drugs, we monitored spheroid size and cell viability (Figs. 5f, g and  S5f, g).

The baseline functional signature of CDD spontaneous calcium oscillations compared to control was used to identify chemicals capable of rescuing such a hypersynchronous phenotype (Fig. 5h). To enable unbiased quantitative analysis of the impact of each treatment on all 17 functional parameters analyzed (including calcium peak height, peak count, and synchronization. See “Methods” for parameters description) we used an aggregate measure of overall system perturbation [57]—parameter recovery (PR) and scalar perturbation (SP). Briefly, PR indicates the proportion of parameters rescued by each compound replicate by dividing the sum of the recovered parameters by the total number of parameters (100% indicates that all parameters are recovered to control boundaries). SP allows for estimating whether a given compound changes the recovery status by an adaptive response. The SP is essentially zero in untreated cells, but as the functional endpoint changes in response to the compound treatment, the SP increases (see “Methods” for details). In other words, the SP and PR allowed us to categorize the screened compounds: the smaller or higher the score, respectively, for SP or PR, the closer the CDD calcium oscillatory activity is from control (the top lead compounds are highlighted in blue; Fig. 5i, j and Table S7). By investigating the correlation between the top 50 compounds, enriched terms included neuronal signaling, protein tyrosine kinase, MAPK, cell cycle, and PI3K/Akt/mTOR (Fig. S5h, i). These findings support the validity of the platform and prompted us to select the top leads.

After combining the analysis of our multiparametric pipeline, we found four chemicals that demonstrated strong therapeutic potential by rescuing CDD’s altered functional phenotype in more than 60% with no effect on cell viability: the hyperpolarization-activated cyclic nucleotide-gated channel blocker Ivabradine (SP ranking = 10, PR ranking = 3), the muscarinic receptor inhibitor Solifenacin (SP ranking = 28, PR ranking = 4), the potent and selective GSK3 inhibitor AZD1080 (SP ranking = 12, PR ranking = 23), and the Notch inhibitor Crenigacestat (SP ranking = 5, PR ranking = 10) (Fig. 5k, l and Tables S7, S8). We further investigated if these compounds could display a dose-dependent action in a similar approach, where the CDD spheres were treated with the top four compounds in six different concentrations (Fig. 5m). Using a nonlinear regression fit, Ivabradine exhibited *EC*_50_ = 0.041 µM with HillSlope = 1.00 and Crenigacestat, *EC*_50_ = 0.883 µM with HillSlope = 39.40.

Next, these lead compounds were investigated for their ability to ameliorate CDD cellular phenotype. Following a similar pharmacological strategy, spheres were treated for three weeks, and cellular migration was evaluated after plating. Ivabradine, Solifenacin, AZD1080, and Crenigacestat were able to rescue outward radial cellular migration defects in CDD (Fig. S5j). Altogether, the platform revealed novel compounds that could be therapeutically useful for CDD and other types of refractory epileptic syndromes.

## Discussion

Epilepsy is one of the most common neurological disorders, categorized mainly by atypical neuronal network electrical activity, leading to recurrent and unpredictable seizures [58]. Treatment, which generally includes medications or sometimes surgery, may eliminate or reduce the frequency and intensity of seizures; however, the mechanism(s) underlying this condition remains unclear. Although much has been done to comprehend the development of epilepsy, the results still insufficient for clinical translation and no effective treatment has emerged yet. In this context, by using a human neurodevelopmental experimental model, we are exploiting a well-defined, mono-genetically driven epilepsy phenotype. Our strategic use of CDKL5-deficient cells as a biological tool to understand its complex phenotypes provides additional insight into the epilepsy field and new compounds for curative or ameliorative therapies.

The human *CDKL5* gene encodes for a protein with a highly conserved serine-threonine kinase domain in its N-terminal, sharing homology to both members of the mitogen-activated protein (MAP) kinase and cyclin-dependent kinase (CDK) families [9]. CDD is one of the lead causes of genetic early-onset epilepsy and is characterized as a devastating neurodevelopmental disorder that has no cure and still lacks effective treatments. Much effort has been made in correlating genotype to symptoms [30], but little is known about its pathophysiology, mainly due to scarce information on CDKL5 protein function and downstream targets. Notably, mouse models for CDD have been controversial regarding the emergence of spontaneous seizures to mirror the human condition [33, 59]. Therefore, the early-onset intractable seizures and the lack of robust mouse models, make the use of iPSC from CDD patients a valid approach to understanding its molecular mechanism and screen novel lead compounds for more effective treatments.

We generated a human stem cell-based model for CDD by reprogramming cells from six patients with different loss-of-function *CDKL5* mutations that lead to an absent or nonfunctional protein. In female patients, we took advantage of the random X-inactivation to identify two pairs of isogenic clones in order to minimize the influence of the individual’s genetic background on the observed phenotypes. Molecular, cellular and functional analysis of cortical organoids and cells derived from patients with mutations in *CDKL5* revealed its specific role in human cells. The CDD neuropathophysiology develops due to multiple alterations: the defective NPC proliferation leading to morphological changes, hyperactivation of the mTOR pathway, and alterations in neuronal connectivity and excitability. In contrast to CDD rodent models showing a decrease in mTORC1 activity [32, 40, 42], our data revealed increased phosphorylation of AKT1S1, EIF3C, RPTOR, LARP1, and rpS6 in CDD neurons. Consistent with mTOR hyperactivation, CDD NPCs have slower proliferation and augmented cell death [60], along with sustained phosphorylation of mTOR downstream targets under amino acid starvation. The enhanced neurite outgrowth observed might be accounted for by the misregulation of chondroitin sulfate degradation observed in the proteomic CDD profile. Moreover, CDD neurons also exhibited significantly longer dendrites, increased spine-like densities, and impaired spine-like motility and synaptogenesis.

The proteomics and phosphoproteomics profiles of CDD cortical neurons point to alterations in connectivity and functionality. Interestingly, the observed hyperactivation of mTOR is a hallmark of epileptic neurological disorders [61]. Thus, we dynamically investigated the network activity of CDD neurons over time. CDKL5-deficient cortical organoids were significantly more active than controls, consistent with QMI data indicating increased AMPAR and mGluR levels in CDD. By evaluating the behavior of individual cells, we confirmed that CDD neurons are more excitable than controls. This hyperexcitability of CDD cortical neurons could explain the occurrence of seizures early in the life of CDD patients [59].

The knowledge of new molecular signatures in CDKL5-deficient neurons prompted us to develop a pharmacological strategy for targeting aberrant integrated neurons into circuits and possibly causing neuronal hyperexcitability in patients. The CDD model was implemented on a validated 3D high-throughput screening (HTS) platform on an industrial scale. We interrogated CDD spheroids at 6 weeks of differentiation by HT calcium imaging assays. A multiparametric analysis, focusing on calcium oscillation frequency and peak irregularities, was used to rank the compound rescue effect. After ensuring that controls were distinguishable and defining a phenotypic “recovery,” two unbiased analysis algorithms were developed (SP and PR) to evaluate the impact of compounds rescuing 17 activity parameters in CDD. Using these independent algorithms and considering the calcium tracing profile, spheroid area and cell viability data, we selected the top lead compounds.

The alkaloid Harmine was one of the top candidate compounds ranking as first and second in the SP and PR, respectively. Notably, this compound was reported to restore CDKL5-dependent synaptic defects in *Cdkl5* knockout mouse neurons [62]. However, we excluded it from our list of top candidates due to its effect on cell survival. Similarly, a mouse model of CDD showed an improvement in development and learning due to pharmacological inhibition of GSK3β [40]. In this context, AZD1080 is a valuable candidate for further studies. Ivabradine was already associated with epilepsy treatment [63], while Crenigacestat was implicated in hippocampal neurogenesis after status epilepticus [64]. All the selected compounds did not significantly reduce cell viability, and some were even able to rescue the size and cellular migration defects of the spheroids. However, our data also suggest caution on the quest for therapeutic compounds. Although the selected lead compounds rescued the combination of the functional phenotypes, Platycodin was the top-ranked compound able to ameliorate the frequency and amplitude of the Calcium peaks with impact on cell viability.

Our study demonstrates the power and necessity of accessing several cellular and functional rescues before in vivo tests. This strategy could reduce downstream failures and expenses in the quest for therapeutic compounds. Therefore, using a human neurodevelopmental model, we revealed potential downstream molecules and pathways affected by *CDKL5* mutation in CDD brain cells and described associated cellular and functional phenotypes related to molecular alterations, opening novel therapeutic opportunities for CDD patients and other refractory epileptic syndromes.

## Methods

### Participant recruitment

Skin biopsies from three female and three male subjects diagnosed with CDD, and their first-degree-related healthy control (mother or father, respectively) were gently donated through a collaboration with the International Foundation for CDKL5 Research. The study was reviewed and approved by the University of California San Diego IRB/ESCRO committee (protocol 141223ZF).

### Cell reprogramming

Fibroblasts from CDD patients and their respective non-affected controls were reprogrammed using non-integrative methods, either by transduction with six episomal plasmid vectors (Sox2, Klf4, Oct3/4, Lin28, p53 shRNA, and L-Myc) or by Sendai virus vector-mediated expression of Oct4, Sox2, Klf4, and c-myc (CytoTune-iPS Kit, Life Technologies), as described elsewhere [65, 66]. iPSCs from each subject were isolated and transferred to feeder-free Matrigel (BD Biosciences) coated plates ~4 weeks after initial transduction. Colonies were propagated in mTeSR1 (Stem Cell Technologies) and manually passaged as small colonies. Standard G-banding karyotype of iPSC clones was performed by the Stem Cell Core Facility at USC (Los Angeles, CA, USA), in collaboration with the Children’s Hospital Los Angeles. Analysis of copy-number variation in iPSC clones was performed by the DRC Genomics and Epigenetics Core at the University of California San Diego.

### Generation of NPCs and 2D neurons

iPSCs were differentiated into NPCs as previously described [45, 46]. Briefly, iPSCs colonies were cultured for 2 days in the presence of DMEM/F12 1:1 with 1x Glutamax (Life Technologies), 1x N2 NeuroPlex (N2; Gemini Bio-products), 1x penicillin-streptomycin (PS; Life Technologies), 10-μm SB431542 (SB; StemGent), and 1-μm dorsomorphin (Dorso; Tocris Biosciences). The colonies were lifted off and kept in suspension, under rotation (95 rpm) for 7 days to form embryoid bodies (EB). EBs were gently disrupted, plated onto Matrigel-coated plates, and cultured in DMEM/F12 1:1 with 1x HEPES, 1x Glutamax, 1x PS, 0.5x N2, 0.5x Gem21 NeuroPlex (Gem21; Gemini Bio-products), supplemented with 20-ng/mL basic fibroblast growth factor (bFGF; Life Technologies) for 7 days. Next, neural rosettes were manually collected, dissociated with Accutase (Thermo Fisher), and NPCs were plated onto poly-L-ornithine/laminin-coated plates. NPCs were expanded in the presence of bFGF and fed every other day. Neural differentiation was promoted by bFGF withdrawn from the medium; ROCK inhibitor (Y-27632; Calbiochem, Sigma-Aldrich) was added at 5 μm for the first 2 days. Medium was changed twice a week, and cells were allowed to differentiate for as long as needed.

### Generation of cortical organoids

Cortical organoids were generated as previously described [48]. Briefly, fully-grown iPSCs colonies were dissociated for ~10 min at 37 °C with Accutase in PBS (1:1), and centrifuged for 3 min at 150 × *g*. The cell pellet was resuspended in mTeSR1 supplemented with 10-µM SB and 1-µM Dorso. Approximately 4 × 10^6^ cells were transferred to one well of a 6-well plate and kept in suspension under rotation (95 rpm) in the presence of 5-µM ROCK inhibitor for 24 h to form free-floating spheres. After 3 days, mTeSR1 was substituted by Media1 [Neurobasal (Life Technologies) supplemented with 1x Glutamax, 1x Gem21, 1x N2, 1x MEM nonessential amino acids (NEAA; Life Technologies), 1x PS, 10-µM SB and 1-µM Dorso] for 7 days. Cells were then maintained in Media2 [Neurobasal with 1x Glutamax, 1x Gem21, 1x NEAA, and 1x PS] supplemented with 20-ng/mL bFGF for 7 days, followed by 7 additional days in Media2 supplemented with 20 ng/mL of FGF2 and 20-ng/mL EGF (PeproTech). Next, cells were transferred to Media3 [Media2 supplemented with 10 µg/mL of BDNF, 10 µg/mL of GDNF, 10 µg/mL of NT-3 (all from PeproTech), 200-µM L-ascorbic acid, and 1-mM dibutyryl-cAMP (Sigma-Aldrich)]. After 7 days, cortical organoids were maintained in Media2 for as long as needed, with media changes every 3–4 days.

### Mycoplasma testing

All cellular cultures were routinely tested for mycoplasma by PCR. Media supernatants (with no antibiotics) were collected, centrifuged, and resuspended in saline buffer. Ten microliters of each sample were used for a PCR with the following primers: Forward: GGCGAATGGGTGAGTAAC; Reverse: CGGATAACGCTTGCGACCT. Only negative samples were used in the study.

### Proteomics and phosphoproteomics analysis

Cell lysates from NPCs, neurons, and cortical organoids were prepared with RIPA buffer or 100-mM TEAB with 1% SDS. After reduction (10-mM TCEP) and alkylation (50-mM chloroacetamide), MeOH/CHCl_3_ precipitation was performed. Pellets were dissolved with 6-M urea in 50-mM TEAB, and LysC/Tryp (Promega) was added at 1:25 (w/w) ratio. After 3–4-h incubation at 37 °C, reaction mixture was diluted with 50-mM TEAB for urea to be <1 M. After overnight digestion, peptides were labeled with TMT 10-plex (Thermo Fisher), followed by quenching with hydroxylamine. All reaction mixtures were pooled together and dried using SpeedVac. One hundred μg of peptides were separated for total protein analyses, and the remaining mixtures were used for phosphoproteomic analyses. After desalting using Pierce peptide desalting spin columns (Thermo Fisher), phosphopeptides were enriched using the High-Select TiO_2_ Enrichment Kit (Thermo Fisher). Resulting eluates were dried in SpeedVac immediately after the enrichment. Peptides to be analyzed in both total and phosphoprotein analyses were fractionated using Pierce High pH Reversed-phase Peptide Fractionation Kit (Thermo Fisher) and then dried in SpeedVac. Dried peptides were dissolved with buffer A (5% acetonitrile, 0.1% formic acid), and each fraction was injected directly onto a 25 cm, 100-μm-ID columns packed with BEH 1.7-μm C18 resin (Waters). Samples were separated at a flow rate of 300 nL/min on nLC 1000 (Thermo Fisher). A gradient of 1–25% buffer B (80% acetonitrile, 0.1% formic acid) over 200 min, an increase to 50% B over 120 min, an increase to 90% B over another 30 min and held at 90% B for a final 10 min of washing was used for 360-min total run time. Column was re-equilibrated with 20 μL of buffer A prior to the injection of sample. Peptides were eluted directly from the tip of the column and nanosprayed directly into the mass spectrometer Orbitrap Fusion by application of 2.8-kV voltage at the back of the column. Fusion was operated in a data-dependent mode. Full MS1 scans were collected in the Orbitrap at 120k resolution. The cycle time was set to 3 s, and within this 3 s, the most abundant ions per scan were selected for CID MS/MS in the ion trap. MS3 analysis with multi-notch isolation (SPS3) [67] was utilized for detection of TMT reporter ions at 60k resolution. Monoisotopic precursor selection was enabled, and dynamic exclusion was used with exclusion duration of 10 s. Tandem mass spectra were extracted from the raw files using RawConverter [68] with monoisotopic peak selection.

The spectral files from all fractions were uploaded into one experiment on Integrated Proteomics Applications (IP2, Ver.5.1.3) pipeline. Proteins and peptides were searched using ProLuCID and DTASelect 2.0 on IP2 against the UniProt *H. sapiens* protein database with reversed decoy sequences (UniProt_Human_reviewed_05-05-2016_reversed.fasta). Precursor mass tolerance was set to 50.0 ppm, and the search space allowed all fully-tryptic and half-tryptic peptide candidates without limit to internal missed cleavage and with a fixed modification of 57.02146 on cysteine and 229.1629 on N-terminus and lysine. Peptide candidates were filtered using DTASelect parameters of -p 1 (proteins with at least one peptide are identified) -y 1 (partial tryptic end is allowed) -pfp 0.01 (protein FDR < 1%) -DM 5 (highest mass error 5 ppm) -U (unique peptide only). Quantification was performed by Census on IP2. The expression value for each protein was calculated by adding the peptide-level reporter ion intensities normalized to total intensity of each channel to remove the variances caused by the different loading amount or labeling efficiency for different channels. For phosphoproteome analysis, search parameters included differential modification of 79.966331 on serine, threonine, and tyrosine, and DTASelect parameters -p 1 -y 1 -pfp 0.01 -DM 5 -m 0 (only phophorylated peptides). To detect the phosphorylation-specific changes, peptide-level reporter ion intensities were normalized to total protein intensities.

### Amino acid starvation

NPCs were grown to 80–90% confluency in complete culture medium before amino acid withdraw. Next, medium was replaced by a glucose-containing starvation buffer, Earle’s Balanced Salt Solution (Thermo Fisher), and the cells were incubated for 10, 30, 60, 120, or 240 min before protein extraction.

### Western blotting

Protein was extracted using the RIPA Lysis and Extraction buffer (Thermo Fisher) containing cOmplete ULTRA mini protease inhibitor (Roche) and PhosSTOP phosphatase inhibitor (Roche). Twenty microgram of protein lysates were separated on a 4–12% Bis-Tris protein gel (Novex), and transferred onto a nitrocellulose membrane (Novex) using the iBlot2 Gel Transfer device (Thermo Fisher). Following blockage with Rockland Blocking Buffer (Rockland), the membrane was incubated with primary antibodies overnight at 4 °C. Next, the membrane was washed five times (5 min each) with 0.1% Tween 20 in PBS and incubated with secondary antibodies for 2 h at room temperature. Antibodies used in this study can be found in Table S9. Odyssey CLx imaging system (Li-Cor) was used for signal detection, and semi-quantitative analysis was performed using Odyssey Image Studio software.

### Immunofluorescence staining

iPSCs, NPCs, and 2D neurons were fixed with 4% paraformaldehyde for 15 min. After three washes with PBS, cells were permeabilized with 0.25% Triton X-100 for 15 min, blocked with 3% bovine serum albumin (BSA) and incubated overnight at 4 °C with primary antibodies diluted in 3% BSA. The following day, cells were washed and incubated with the secondary antibodies for 1 h. Antibodies used in this study can be found in Table S9. For nuclei staining, DAPI solution (1 μg/mL) was used. The slides were mounted using ProLong Gold antifade reagent and analyzed under a fluorescence microscope (Z1 Axio Observer Apotome, Zeiss).

### Synaptic puncta quantification

After 8 weeks of differentiation, 2D neurons were fixed and stained. The number of pre-synaptic VGLUT1+ and post-synaptic HOMER1+ puncta co-localization was blindly quantified. Only puncta overlapping MAP2+ processes were scored. Images were taken randomly from two independent experiments.

### DNA fragmentation analysis

NPCs were harvested to single-cell suspension in PBS, fixed by addition of 70% ethanol and stored for 24 h at 4 °C. Next, cells were washed with PBS, resuspended in DAPI staining solution (0.1% (v/v) Triton X-100, 1-µg/mL DAPI in PBS) and incubated for 5 min at 37 °C. Samples were analyzed on the NC-3000 Advanced Image Cytometer (Chemometec), using the preoptimized DNA Fragmentation Assay. The amount of high molecular weight DNA retained in the cells was quantified in order to detect apoptotic cells with fragmented DNA (subG1 population).

### Caspase assay

Caspase activity was assessed using the Green FLICA Caspases 3 & 7 Assay Kit (ImmunoChemistry Technologies, LLC) according to manufacturer’s protocol. Briefly, NPCs were harvested to single-cell suspension, washed with PBS, and stained with 1X carboxyfluorescein Fluorochrome Inhibitor of Caspase Assay (FAM-FLICA) reagent, 10-µg/mL Hoechst 33342 and 10-µg/mL propidium iodide (PI). Samples were analyzed on the NC-3000 Advanced Image Cytometer (Chemometec) using the preoptimized Caspase Assay. PI stained the nonviable cell population whereas FAM-FLICA stained cells with caspase activity for apoptosis analysis.

### Proliferation assay

NPC proliferation was assessed by cell counting. Briefly, a pre-determined number of NPCs was plated onto poly-L-ornithine/laminin-coated plates (day 0). After 4 h, the plates were transferred to a Viva View FL Incubator Microscope (Olympus), and allowed to acclimatize for 30 min (T0). Next, the cellular proliferation was monitored and contrast-phase images were taken after 48 h (T48; day 2). The images were processed, and the number of cells at T0 and T48 was determined using the Cell Counter plugin on the Fiji platform. The difference between days 2 and 0 was used to estimate the NPC proliferation rate.

### Mitochondrial depolarization

Disruption of the mitochondrial transmembrane potential (Dym) is usually associated with early stages of apoptosis. We measured Dym in NPCs using the cationic dye JC-1. Briefly, NPCs were harvested to single-cell suspension in PBS containing 2.5 μg/mL of a JC-1 solution, and cells were incubated for 10 min at 37 °C. After washes with PBS, DAPI (1 μg/mL) was added for nuclei staining. Samples were analyzed on the NC-3000 Advanced Image Cytometer (Chemometec) using the preoptimized Mitochondrial Potential Assay. At high concentrations, JC-1 forms aggregates and become red fluorescent, while in apoptotic cells the mitochondrial potential collapses and JC-1 localizes to the cytosol in its monomeric green fluorescent form. The number of cells with collapsed mitochondrial membrane potential was quantified and the mitochondrial depolarization estimated as a decrease in the red/green fluorescence intensity ratio.

### Neuronal spine-like dynamics

Dendrites from 8-week-old neurons stably transfected to express enhanced GFP by Synapsin1 promoter self-inactivating lentivirus were recorded using a Z1 Axio Observer Apotome (Zeiss). Images were taken every 30 s for 1 h, and analyzed using the NeuronJ plugin on the Fiji platform. Only neurons that displayed at least two visible neurites at *t* = 0 and had changes in spine dynamics during the 60-min time course were analyzed.

### Cellular migration

Three-week old spheres were treated for 3 additional weeks with 1 µM of selected compounds. Cellular migration was evaluated 8 days after spheres were plated onto poly-L-ornithine/laminin-coated plates. The outward radial migration was measured using NeuronJ plugin on the Fiji platform.

### Quantitative multiplex co-immunoprecipitation (QMI)

QMI analysis was performed as previously described [53, 54]. Briefly, cortical organoids were homogenized in lysis buffer [150-mM NaCl, 50-mM Tris (pH 7.4), 1% NP-40, 10-mM NaF, 2-mM sodium orthovanadate + protease/phosphatase inhibitor cocktails (Sigma)] using a glass tissue homogenizer, incubated for 15 min, centrifuged at high speeds to remove nuclei and debris, and protein concentration was determined using a Pierce BCA Kit (Thermo Fisher). A master mix containing equal numbers of each antibody-coupled Luminex bead class was prepared and distributed into post-nuclear cell lysate samples in duplicate. Protein complexes were immunoprecipitated from samples containing equal amounts of protein overnight at 4 °C, washed twice in an ice-cold Fly-P buffer [50-mM tris (pH 7.4), 100-mM NaCl, 1% BSA, and 0.02% sodium azide], and distributed into as many wells of a 96-well plate as there were probes, on ice. Biotinylated detection antibodies were added and incubated for 1 h, with gentle agitation at 500 rpm in a cold room (4 °C). Following incubation, microbeads and captured complexes were washed three times in the Fly-P buffer using a Bio-Plex Pro II magnetic plate washer in a cold room. Microbeads were then incubated for 30 min with streptavidin-PE on ice, washed three times, and resuspended in 125 μL of an ice-cold Fly-P buffer. Fluorescence data were acquired on a customized, refrigerated Bio-Plex 200 instrument according to the manufacturer’s recommendations.

Data preprocessing and inclusion criteria XML output files were parsed to acquire the raw data for use in MATLAB while XLS files were used for input into R statistical packages. For each well from a data acquisition plate, data were processed to (i) eliminate doublets on the basis of the doublet discriminator intensity (>5000 and <25,000 arbitrary units; Bio-Plex 200), (ii) identify specific bead classes within the bead regions used, and (iii) pair individual bead PE fluorescence measurements with their corresponding bead regions. This processing generated a distribution of PE intensity values for each pairwise protein co-association measurement. ANC adaptive nonparametric analysis with empirical alpha cutoff (ANC) [69] was used to identify high-confidence, statistically significant differences (corrected for multiple comparisons) in bead distributions on an individual interaction basis. ANC was conducted as described elsewhere [54].

We required that hits be present in at least six of eight replicates at an adjusted *p* < 0.05. The *α*-cutoff value required per experiment to determine statistical significance was calculated to maintain an overall type I error of 0.05 (adjusted for multiple hypothesis testing with Bonferroni correction), with further empirical adjustments to account for technical errors. CNA: bead distributions used in ANC were collapsed into a single median fluorescent intensity (MFI), which was averaged across duplicate samples and input into the WGCNA package for R [70]. Data were filtered to remove weakly detected interactions (“noise,” MFI < 100), and batch effects were removed using the COMBAT function for R [54], with “experiment number” as the “batch” input. Post-Combat data were log2-transformed prior to CNA analysis. Closely related protein co-associations were assigned to arbitrary color-named modules by the WGCNA program. Modules whose eigenvectors significantly (*P* < 0.05) correlated with the genotype were considered significantly as described previously [54, 69] to produce a high-confidence set of interactions that were both individually significantly different in comparisons between experimental groups, and that belonged to a larger module of co-regulated interactions that was significantly correlated with experimental group. Hierarchical clustering was performed using pvlcust in R [71].

### NPC transplantation

Human iPSC-derived NPCs were stably transfected to express EGFP by Synapsin1 promoter self-inactivating lentivirus. Ten to twenty thousand cells were injected per site, 1 mm from the midline between the Bregma and Lambda and 1–2-mm deep into the cortex and striatum of newborns immunosuppressed NOD/SCID mice. Briefly, newborns (P0–P2) were anesthetized by hypothermia and then placed in a contoured Styrofoam mold. Two microliters of NPCs were injected into both hemispheres using a 5-ml Hamilton syringe with a 32-gauge needle. After 6 months, injected animals were anesthetized and perfused. Entire brains were sliced using a cryomicrotome, and immunohistochemical analysis was carried out on free-floating mice brain slices to identify and evaluate the efficiency of the transplantation.

### Postmortem brain specimens and cortical sampling

Four postmortem brains that were gender, age, and hemisphere-matched were used. Specifically, postmortem brain tissue from a 5-year-old female CDD patient with a Pro719CysfsX66 mutation and, a 30-year-old female CDD patient with a deletion comprehending exons 1–3 in the *CDKL5* gene; and two female individuals with no described genetic alteration (control), respectively at 6- and 30-year-old. All brain specimens were harvested within a postmortem interval of 15–36 h and had been immersed and fixed in 10% formalin for <3 years. For the purpose of the present experiments, samples were obtained from anatomically well-identified cortical areas in a consistent manner across specimens, comprehending the primary somatosensory cortex (Brodmann area 3), the primary motor cortex (Brodmann area 4) and the secondary visual area (Brodmann area 18). Details about the tissue-processing protocol is provided online by the NIH NeuroBioBank. We focused specifically on these parts of the cortex because pathologies in dendritic morphology in these areas have been reported in other neurodevelopmental disorders [46, 72–74]. In addition, pyramidal neurons in the selected areas reach their mature-like morphology early in development and start displaying dendritic pathologies sooner than high integration areas, such as the prefrontal cortex, allowing for a comparison of postmortem findings with iPSC-derived neurons in early stages of development [75, 76]. Samples were obtained from Harvard Brain Tissue Resource Center, the University of Maryland Brain and Tissue Bank, and the University of Miami Brain Endowment Bank, which are Brain and Tissue Repositories of the NIH NeuroBioBank.

### Postmortem brain tissue processing

Processing and staining of brain tissue samples were performed by Neurodigitech LLC (San Diego, CA) using the semi-rapid Golgi technique [77]. Briefly, specimens were immersed in a solution of 1% silver nitrate for 10 days. Blocks were then sectioned on a vibratome, perpendicular to the pial surface, at a thickness of 110–120 μm. Golgi sections were cut into 100% ethyl alcohol and transferred briefly into toluene, mounted onto glass slides, and cover-slipped.

### Golgi-impregnated neurons

Neurons included in the morphological analysis did not display degenerative changes [78]. The morphological analysis was performed on pyramidal neurons located in cortical layers V/VI, with fully impregnated soma, apical dendrites with present oblique branches, and at least two basal dendrites with second/third order segments. To minimize the effects of cutting on dendritic measurements, we included neurons with cell bodies located near the center of 120-μm thick histological sections, with natural terminations of higher-order dendritic branches present where possible [79, 80]. Inclusion of the neurons completely contained within 120-μm sections biases the sample toward smaller neurons, leading to the underestimation of dendritic length [81]; therefore, we applied the same criteria blinded across all control and CDD specimens, and we thus included the neurons with incomplete endings if they were judged to otherwise fulfill the criteria for successful Golgi impregnation. All neurons were oriented with apical dendrite perpendicular to the pial surface; inverted pyramidal cells as well as magno-pyramidal neurons were excluded from the analysis.

### Postmortem brain neuronal tracing

Neuronal morphology was quantified along *x*-, *y*-, and *z*-coordinates using OPTIMAS Bioscan software (Media Cybernetics Inc., MD, USA) connected to a Zeiss Axio scope system, equipped with 100×/1.25 oil Plan Fluor objective and a CCD camera (Hamamatsu, ORCA-Flash4.0 V3), motorized *X-*, *Y*-, and *Z*-focus for high-resolution image acquisition and digital quantitation. Tracings were conducted on both apical and basal dendrites, and the results reflect summed values for both types of dendrites per neuron. Following the recommendation that the applications of Sholl’s concentric spheres for the analysis of neuronal morphology are not adequate when neuronal morphology is analyzed in three dimensions [81], we conducted dendritic tree analysis with the following measurements [79, 80]: (1) soma area—cross sectional surface area of the cell body; (2) dendritic length—summed total length of all dendrites per neuron; (3) dendrite number—number of dendritic trees emerging directly from the soma per neuron; (4) dendritic segment number—total number of segments per neuron; (5) dendritic spine/protrusion number—total number of dendritic spines per neuron; (6) dendritic spine/protrusion density—average number of spines/20 μm of dendritic length; and (7) branching point number—number of nodes (points at the dendrite where a dendrite branches into two or more) per neuron. Dendritic segments were defined as parts of the dendrites between two branching points—between the soma and the first branching point in the case of first order dendritic segments, and between the last branching point and the termination of the dendrite in the case of terminal dendritic segments. Since the long formalin-fixation time may result in degradation of dendritic spines, spine values may be underestimated and are thus reported here with caution. All of the tracings were accomplished blind to brain region and diagnostic status.

### iPSC-derived neuronal tracing

The iPSC-derived samples consisted of EGFP-SYN1-positive 8-week-old neurons with pyramidal- or ovoid-shaped soma and at least two branched neurites (dendrites) with visible spines/protrusions. Protrusions from dendritic shaft, which morphologically resembled dendritic spines in postmortem specimens, were considered and quantified as dendritic spines in iPSC-derived neurons. The neurites were considered dendrites based on the criteria applied in postmortem studies: (1) thickness that decreased with the distance from the cell body; (2) branches emerging under acute angle; and (3) presence of dendritic spines. Only EGFP-positive neurons with dendrites displaying evenly distributed fluorescent stain along their entire length were considered. In addition, neurons included in the analysis had to exhibit the nuclei co-stained with CTIP2, indicative of layer V/VI neurons. The morphology of the neurons was quantified along *x*-, *y*-, and *z*-coordinates using Neurolucida v.9 software (MBF Bioscience, Williston, VT) connected to a Nikon Eclipse E600 microscope with 40× oil objective. No distinction was made between apical and basal dendrites, and the results reflect summed length values of all neurites/dendrites per neuron, consistent with what was done for the postmortem neurons. The same set of measurements used in the analysis of Golgi-impregnated neurons was applied to the analysis of iPSC-derived neurons, and all tracings were accomplished blind to the genotype.

### MEA recording

Six-week-old cortical organoids were plated per well in poly-L-ornithine/laminin-coated 12-well MEA plates (Axion Biosystems). Cells were fed twice a week, and measurements were collected 24 h after the medium was changed, once a week, starting at 2 weeks after plating (8 weeks of organoid differentiation). Recordings were performed using a Maestro MEA system and AxIS Software Spontaneous Neural Configuration (Axion Biosystems) with a customized script for band-pass filter (0.1-Hz and 5-kHz cutoff frequencies). Spikes were detected with AxIS software using an adaptive threshold crossing set to 5.5 times the standard deviation of the estimated noise for each electrode (channel). The plate was first allowed to rest for 3 min in the Maestro device, and then 3 min of data were recorded. For the MEA analysis, the electrodes that detected at least 5 spikes/min were classified as active electrodes using Axion Biosystems’ Neural Metrics Tool. Bursts were identified in the data recorded from each individual electrode using an inter-spike interval (ISI) threshold requiring a minimum number of five spikes with a maximum ISI of 100 ms. At least ten spikes under the same ISI with a minimum of 25% active electrodes were required for network bursts in the well. The synchrony index was calculated using a cross-correlogram synchrony window of 20 ms. Independent experiments were performed with three cell lines with at least three technical replicates.

### Cellular electrophysiology

Whole-cell patch-clamp recordings were performed in cultured human iPSC-derived neurons at room temperature (~20 °C), as described previously [44, 82]. The extracellular solution for patch-clamp experiments contained the following: 130-mM NaCl, 3-mM KCl, 1-mM CaCl_2_, 1-mM MgCl_2_, 10-mM HEPES, and 10-mM glucose; pH 7.4 with 1-M NaOH (∼4 mM Na^+^ added). The internal solution for patch electrodes contained the following: 138-mM K-gluconate, 4-mM KCl, 10-mM Na_2_-phosphocreatine, 0.2-mM CaCl_2_, 10-mM HEPES (Na^+^ salt), 1-mM EGTA, 4-mM Mg-ATP, 0.3-mM Na-GTP; pH 7.4 with 1-M KOH (∼3-mM K^+^ added). The osmolarity of all solutions was adjusted to 290 mOsm. Electrodes for electrophysiological recording were pulled on a Flaming/Brown micropipette puller (Model P-87, Sutter Instrument) from filamented borosilicate capillary glass (1.2-mm OD, 0.69-mm ID, World Precision Instruments). The electrode resistances were 3–6 MΩ for whole-cell recordings. Patch-clamp experiments were performed with an Axon CV-4 headstage, Axopatch 200A amplifier and DigiData 1322A (Molecular Devices). Recordings were digitized at 10 kHz and low-pass filtered at 2 kHz. The spontaneous excitatory synaptic currents were recorded with the holding membrane potentials of −60 mV and last 3–5 min for each neuron. Evoked APs were measured from −60 mV under current-clamp configuration. Data were all corrected for liquid junction potentials (10 mV). Voltage-clamp synaptic currents (sEPSCs) were analyzed using Mini Analysis (Synaptosoft) and other electrophysiological results were analyzed using pCLAMP 10 software (Molecular Devices). The electrophysiological experiments and analyses were performed in a blinded manner to avoid bias.

### CDD HTS platform development

An HTS platform for CDD was developed by StemoniX Inc. Briefly, StemoniX’s HTS system relies on spheroids derived from human iPSCs that comprise a balanced culture of cortical neurons and astrocytes. These spheroids display quantifiable, robust, and uniform spontaneous calcium oscillations, which correlated with synchronous neuronal activity [55]. The CDD HTS platform uses cells derived from CDD1 and Control1 lines.

### Chemical library and drug treatment

The compound library used in this study comprises a unique collection of 1112 compounds with biological activity used for neurologic research and associated assays; of which two-thirds are FDA-approved drugs (SelleckChem, Houston, TX, USA). Three-week-old CDD spheroids received chronic treatment three times per week, during 3 weeks. All compounds were tested at 1 µM in three technical replicates; vehicle controls (DMSO or water) were included in multiple replicates. The dose–response assay comprehended six concentrations ranging from 0.0003 to 1 µM, in four technical replicates.

### HT calcium oscillation assay

To assess the intracellular calcium oscillations in CDD and control spheroids, cells were incubated with the FLIPR Calcium 6 Kit (Molecular Devices LLC, San Jose, CA, USA) as previously described [55]. Briefly, cells were pre-loaded with Calcium 6 Dye for 2 h prior to the recording. The peaks observed correlate with synchronous neural activity in the spheroids. The kinetics of intracellular calcium oscillations was determined using the FLIPR Tetra High-Throughput Cellular Screening System (Molecular Devices LLC): the fluorescence intensity was set at 515–575 nm following excitation at 470–495 nm for 10 min at a frequency of 3 Hz; the exposure time per read was 0.4 s, the gain was set to 30, and the excitation intensity was set to 40%. The instrument temperature was kept at 37 °C.

### HT cell viability

To determine any cytotoxic effect of the drug treatment on the neural culture, the CellTiter Glo 3D Cell Viability Assay (Promega, Madison, WI, USA) was performed according to the manufacturer’s instructions. Briefly, following the intracellular calcium oscillation recording, the spheroids were washed with PBS for removal of the calcium dye. Next, cells were incubated with CellTiter Glo Reagent for 30 min at room temperature and the number of viable cells was estimated based on the amount of ATP present in the culture. The luminescent signal was captured using a PHERAstar FSX Microplate Reader (BMG Labtech, Ortenberg, Germany).

### HT 3D imaging for size measurement

To determine the size of CDD and control spheroids, contrast-phase images from the 384-well plates were weekly acquired using the ImageXpress Microscope-Micro Confocal System (Molecular Devices LLC). The instrument temperature was kept at constant 37 °C during image acquisition.

### HT screening analysis

A multiparametric analysis of representative descriptors of the intracellular calcium oscillation was performed using StemoniX’s proprietary Assay AnalytiX Software. The features used include: (1) peak count, (2) average (Avg) peak height (amplitude), (3) peak height standard deviation (s.d.), (4) Avg peak width, (5) peak width s.d., (6) Avg peak spacing, (7) peak spacing s.d., (8) Avg peak rise time, (9) peak rise time s.d., (10) Avg peak decay time, (11) peak decay time s.d., (12) Class 1 peak count, (13) Class 2 peak count, (14) Class 3 peak count, (15) singular peak count, (16) irregular peak count, and (17) subpeak count. For peak classification, the relative height of each individual peak within the signal is classified based on percentage based binning of the max peak height into three classes, thus providing a count of peaks across the variation activation levels achieved by the system. Singular peaks are defined as uninterrupted calcium oscillations, meaning the activity from trough to peak and back to trough is continuous, without additional peaks occurring during the ascent or descent of the oscillation. Irregular defines the peaks which have been interrupted by such events, and subpeaks are the number of the interrupting peak events within a signal. To determine the Scalar Perturbation (SP), the data were normalized by dividing raw parameters by the median values of controls from the same plate. Each parameter was standardized by *z*-score of normalized and log-transformed control values. Next, calculating the Euclidean norm of selected parameters provides a SP for each replicate, which was then averaged among the replicates. For Parameter Recovery (PR), boundaries of rescue criteria were set using the parameter values extracted from vehicle control signals. These boundaries were used to calculate the number of rescued parameters for a given compound. Using the number of rescued parameters out of the total parameters considered, a percentage based PR was calculated and averaged across compound replicates.

To determine the correlation between compounds, pathways, and genes, we used the databases NCATS (National Center for Advancing Translational Sciences)–Inxight: Drugs portal (version 1.1, https://drugs.ncats.io/), CTD (Comparative Toxicogenomics Database) [83], KEGG (Kyoto Encyclopedia of Genes and Genomes), DGIdb (Drug Gene Interaction Database) [84]. NCATS Inxight: drugs is a comprehensive portal for drug development information, and contains information on ingredients in medicinal products, including US-approved drugs, marketed drugs, and investigational drugs. CTD is a literature-based and manually curated associations between chemicals, gene products, phenotypes, diseases and environmental exposures. KEGG is a biological encyclopedia for the understanding of high-level functions and utilizes of a biological system and its parts (cell, molecules, genes) and, DBIdb is a drug-gene interaction database, composed by consolidated interactions and druggable genes that were extracted from peer-reviewed manuscripts, databases, and web resources.

### Statistical analysis

Data are presented as mean ± s.e.m., unless otherwise indicated. No statistical method was used to predetermine the sample size. The statistical analyses were performed using GraphPad Prism v6; two-tailed Mann–Whitney *U* test or unpaired *t*-test with multiple-comparison correction was used as indicated. Significance was defined as *p* < 0.05(*), *p* < 0.01(**), *p* < 0.001(***), or *p* < 0.0001(****). Blinding was used for comparing affected and control samples.

## Supplementary information

Supplemental information

Supplemental figure 1

supplemental figure 2

Supplemental Figure 3

Supplemental Figure 4

Supplemental Figure 5

Supplemental Table S2

supplemental Table S3

Supplemental Table S4

Supplemental Table S5

Supplemental Table S6

Supplemental Table S7

Supplemental Table S8

## Data Availability

All data and/or analyses generated during the current study are available from the corresponding author upon reasonable request.
