# Supplementary material for: Altered network and rescue of human neurons derived from individuals with early-onset genetic epilepsy
Source: Mol Psychiatry. 2021 Apr 22;26(11):7047–68. doi: 10.1038/s41380-021-01104-2 (PMC8531162; doi:10.1038/s41380-021-01104-2)
Supplement: Supplementary file 1 — Supplemental information [file 41380_2021_1104_MOESM1_ESM.docx]

**SUPPLEMENTARY FIGURE LEGENDS**

**Supplementary Fig. S1 | Characterization of CDD and control iPSC lines. a**, Information of the subjects included in this study. **b**, Representative immunofluorescence images of markers for pluripotency in CDD and control iPSCs. Scale bar, 100 μm. **c**, Expression of pluripotency gene markers in fibroblasts, ESC and iPSCs. In the Euclidian matrix distance-based heatmap and hierarchical clustering-based dendrogram (left), the variability of expression among samples is indicated by Z-score, varying from green (negative variation) to red (positive variation). **d**, Representative images of male and female karyotypes of CDD cell lines assessed by G-banding analysis. **e**, Representative graphical outputs of SNP-based arrays for copy number variation analysis in male and female iPSC lines. **f**, Representative images of iPSCs immunostained with antibodies for H3K27me3 (red). The single punctate foci of H3K27me3 in female cells (top) indicates the presence of an X chromosome inactivation (Xi), while no apparent nuclear accumulation of H3K27me3 is seen in male cells (bottom). **g**, RNA sequence chromatogram showing the *CDKL5* nucleotide changes in CDD lines compared to controls.

**Supplementary Fig. S2 | Proteomic and phosphoproteomic characterization of CDD neural cell lines. a** and **b**, Gene expression levels of *CDKL5* (**a**) and CDKL5 protein content (**b**) in control cells at different stages of differentiation (iPSC, NPC and 6-week-old neurons). **c,** CDKL5 protein content observed in 6-week-old neurons derived from CDD patients and control individuals, compared to post-mortem human cortical brain lysate. **d**, No differences are observed in the nucleus area and number of MAP2+ cells of 2-month-old CDD organoids compared to control. **e**, Volcano plots illustrating the global proteomic and phosphoproteomic changes in 2-month-old CDD organoids compared to control. All the quantified proteins were plotted (Control, *n =* 4 cell lines; CDD, *n =* 4 cell lines). **f**, Biological functions and diseases showing a significant overlap with phosphorylation changes in 6-week-old CDD neurons (Control, *n =* 5 cell lines; CDD, *n =* 5 cell lines). **g**, Relative phosphorylation of representative microtubule associated proteins in 6-week-old neurons. Relative ratios of phosphopeptide intensity normalized to non-phosphoprotein intensity are shown (Control, *n =* 5 cell lines; CDD, *n =* 5 cell lines; unpaired *t*-test, **P* < 0.05, ***P* < 0.01, ****P* < 0.001). **h**, Site-specific phosphorylation changes in proteins related with microtubule dynamics in CDD neurons (Control, *n =* 5 cell lines; CDD, *n =* 5 cell lines). **i**, TMT reporter ion intensity (normalized to total intensity of each channel) showing that GABA receptor proteins are downregulated in 6-week-old CDD neurons (Control, *n =* 5 cell lines; CDD, *n =* 5 cell lines). **j**, Phosphorylation changes associated with mTOR pathway in CDD neurons. Phosphoproteins increased in CDD neurons are highlighted in red (diagram adopted from IPA). **k**, Western blot quantification of proteins at downstream of mTOR signaling pathway in CDD and control neurons lysates (Control, *n =* 6 cell lines; CDD, *n =* 6 cell lines; two-tailed Mann–Whitney U test). Data are shown as mean ± s.e.m. Individual values are indicated by dots where each symbol represents a subject. CDD and related controls share the same symbol and filled symbols represent isogenic cells. **l**, Time-dependent responses of CDD and control NPCs upon amino acid starvation. The Western blot band intensity was quantified using a LI-COR imaging system and the phosphorylated/total ratio was calculated for each target considering each CDD sample and its related control.

**Supplementary Fig. S3 | Morphometric analysis of CDD neurons. a**, CDD neurons show no differences in the number of dendrites or branching points compared to control *in vitro* (Control, *n =* 64 and 14, respectively for number of dendrites and branching points, 5 cell lines each; CDD, *n =* 64 and 13, respectively for number of dendrites and branching points, 5 cell lines each; minimum of three technical replicates per cell line; two-tailed Mann–Whitney U test). **b**, Top, Representative immunofluorescence images of human neurons transplanted into the mouse brain. Scale bar, 100 μm. Middle, the spine density and complexity of human neurons transplanted into the mouse brain increase over time. Bottom, Mouse brain sections with HuNu-positive cells (red) from anterior to posterior. Scale bar, 1000 µm. Representative immunofluorescent images of transplanted human neurons after 6-month differentiation. **c**, No significant differences were observed in cell body area, number of dendrites, branching points or dendritic segment between CDD and control neurons at 6 months after transplantation into a mouse brain (Control, *n =* 10, 2 cell lines; CDD, *n =* 12, 2 cell lines; minimum of five technical replicates per cell line). **d**, Representative images of post-mortem cortical layer V/VI neurons from CDD and control subjects using Golgi staining. Scale, 25 μm. **e**, Morphometric analyses of post-mortem cortical layer V/VI pyramidal neurons from different Broadman areas (BA). Post-mortem tissues obtained from CDD and control subjects at 5- and 6-year-old, respectively. **f**, Morphometric analyses of post-mortem cortical layer V/VI pyramidal neurons obtained from 30-year-old subjects. Note the differences in cell body area between CDD and control depending on the BA under evaluation. **g**, Left, representative contrast-phase images of cortical organoids at different stages of development. Scale bar, 200 μm. Right, diameter measurements performed at the maturation stage (Control, *n =* 165, 2 cell lines; CDD, *n =* 217, 2 cell lines; minimum of seventy technical replicates per cell line; two-tailed Mann–Whitney U test, *****P* < 0.0001). **h**, Left, representative contrast-phase images of neural cell migration from cortical organoids plated at early stages of development. Scale bar, 200 μm. Right, migration distances measured five days after plating (Control, *n =* 25, 3 cell lines; CDD, *n =* 20, 3 cell lines; minimum of four technical replicates per cell line; two-tailed Mann–Whitney U test, *****P* < 0.0001). Data are shown as mean ± s.e.m. Individual values are indicated by dots where each symbol represents a subject. CDD and related controls share the same symbol and filled symbols represent isogenic cells.

**Supplementary Fig. S4 | Synaptogenesis and electrophysiological activity of CDD neurons.** **a**, Western blot showing the expression levels of pre- and post-synaptic proteins (Synapsin 1 and PSD-95, respectively) in 8-week-old CDD neurons and controls. (Control, *n =* 6 cell lines; CDD, *n =* 6 cell lines; each cell line was derived from a different subject). **b**, Principal component plot of protein interaction network matrices showing two technical replicates run in parallel (connected by lines) from each of two CDD and control (CTL) lines. The QMI experiment was repeated twice (R1 and R2) to illustrate batch and background effects (Control, *n* = 8, 2 cell lines; CDD, *n* = 8, 2 cell lines; four technical replicates per cell line, two independent experiments). **c**, No differences were observed in cell capacitance (Cm), action potential (AP) amplitude or width in CDD neurons compared to control by whole-cell patch clamping (Cm: Control, *n =* 20, 2 cell lines; CDD, *n =* 48, 3 cell lines; AP amplitude: Control, *n =* 14, 2 cell lines; CDD, *n =* 39, 3 cell lines; AP ½ width: Control, *n =* 14, 2 cell lines; CDD, *n =* 29, 3 cell lines; minimum of four technical replicates per cell line; two-tailed Mann–Whitney U test). **d**, Current densities of and *I*_KA_ (left) and Na current densities (right) for CDD and control neurons (***P* < 0.01 and *****P* < 0.0001). **e**, Spontaneous excitatory postsynaptic current (sEPSCs) amplitude in CDD and control neurons (*****P* < 0.0001). Data are shown as mean ± s.e.m.; individual values are indicated by dots where each symbol represents a subject; CDD and related controls share the same symbol; filled symbols represent isogenic cells. **f**, Network activity of CDD and control cortical organoids over time, characterized by different parameters using the MEA (Control, *n =* 6, 2 cell lines; CDD, *n =* 6, 2 cell lines; three technical replicates per line; unpaired *t*-test, each row was analyzed individually without assuming a consistent SD, **P* < 0.05). Data are shown as mean ± s.e.m.

**Supplementary Fig. S5 | Characterization of CDD high throughput screening platform.** **a** and **b**, Box plot showing the differences in peak count (**a**) and amplitude (**b**) for CDD and control calcium oscillation activity in spheroids. DMSO (0.001%) and water were used as vehicle for the screening compounds (Control, *n =* 72, 1 cell line; CDD, *n =* 408, 1 cell line). Data are shown as mean ± s.d. **c** and **d**, Principal component analysis (PCA) was used to compare the distribution of CDD and control calcium oscillation activity on the basis of peak count and amplitude. DMSO (0.001%) (**c**) and water (**d**) were used as vehicle for the screening compounds (Control, *n =* 72, 1 cell line; CDD, *n =* 408, 1 cell line). **e**, Changes in the peak classification of CDD and control calcium oscillations over time. **f**, Representative contrast-phase images of CDD and control spheroids over time. **g**, Area of control and CDD-untreated spheroids over time (Control, *n =* 48, 1 cell line; CDD-untreated, *n =* 88, 1 cell line). Data are shown as mean ± s.d. **h**, Top enriched terms related to the top 50 compounds able to rescue more than 60% of CDD calcium oscillation activity. **i**, Overlapping interaction on the mechanism of action (MOA) of the top 50 compounds.

**j,** Cellular migration distances observed in CDD spheres after treatment (Control, *n =* 107, 3 cell lines; CDD untreated, *n =* 120, 3 cell lines; CDD treated, *n* = 90 for each individual treatment, 3 cell lines; minimum of 30 technical replicates per cell line; one-way ANOVA analysis, *****P* < 0.0001). Data are shown as mean ± s.e.m.

**SUPPLEMENTARY TABLES**

**Supplementary Table S1 |** Description of cell lines used in each experiment.

| Assay | CTL1 | CTL2 | CTL3 | CTL4 | CTL5 | CTL6 | CDD1 | CDD2 | CDD3 | CDD4 | CDD5 | CDD6 |
| --- | --- | --- | --- | --- | --- | --- | --- | --- | --- | --- | --- | --- |
| *CDKL5* gene sequencing | X | X | X | X | X |  | X | X | X | X | X |  |
| Microarray for Pluripotency | X |  |  | X | X | X | X |  |  | X | X | X |
| SNP-based Array for CNV (karyotyping) | X | X | X | X | X | X | X | X | X | X | X | X |
| G-banding Karyotyping | X |  | X |  | X | X | X |  | X |  | X | X |
| iPSC Staining | X | X | X | X | X | X | X | X | X | X | X | X |
| X Chromosome Inactivation Staining | X | X | X | X |  |  | X | X | X | X |  |  |
| NPC Staining | X | X | X | X | X | X | X | X | X | X | X | X |
| Neuronal Staining | X | X | X | X | X | X | X | X | X | X | X | X |
| NPC Proteomics and Phosphoproteomics | X |  | X | X | X |  | X |  | X | X | X |  |
| 2D Neurons Proteomics and P-proteomics | X | X | X | X | X |  | X | X | X | X | X |  |
| Organoid Proteomics and P-proteomics | X | X | X | X |  |  | X | X | X | X |  |  |
| Proliferation | X |  |  | X | X |  | X |  |  | X | X |  |
| Cell Cycle | X |  | X | X | X |  | X |  | X | X | X |  |
| DNA Fragmentation | X | X | X | X |  |  | X | X | X | X |  |  |
| Caspase Positive Cells | X |  | X | X | X |  | X |  | X | X | X |  |
| Mitochondrial Potential | X |  | X | X | X |  | X |  | X | X | X |  |
| CDKL5 Western Blot | X | X | X | X | X | X | X | X | X | X | X | X |
| PS6/S6 Western Blot | X | X | X | X | X | X | X | X | X | X | X | X |
| PS6K/S6K Western Blot | X | X | X | X | X | X | X | X | X | X | X | X |
| P4E-BP1/4E-BP1 Western Blot | X | X | X | X | X | X | X | X | X | X | X | X |
| Amino Acid Starvation | X |  |  | X | X |  | X |  |  | X | X |  |
| Nuclei Size Evaluation | X |  | X | X |  |  | X |  | X | X |  |  |
| MAP2 Positive Cell Counting | X |  | X |  |  |  | X |  | X | X |  |  |
| Spine-like Motility | X |  |  | X | X |  | X |  |  | X | X |  |
| Spine-like Density | X | X | X | X | X |  | X |  |  | X | X |  |
| In vitro Neuronal Tracing and Sholl Analysis | X | X | X | X | X |  | X | X | X | X | X |  |
| In vivo Neuronal Tracing and Sholl Analysis | X |  |  | X |  |  | X |  |  | X |  |  |
| Cortical Organoid Size | X |  |  | X |  |  | X |  |  | X |  |  |
| Cellular Migration | X | X |  | X |  |  | X | X |  | X |  |  |
| Cellular Migration after Drug Treatment | X |  |  | X | X |  | X |  |  | X | X |  |
| Synapsin1 Western Blot | X | X | X | X | X | X | X | X | X | X | X | X |
| PSD95 Western Blot | X | X | X | X | X | X | X | X | X | X | X | X |
| Co-localized Puncta Quantification | X | X | X | X | X |  | X | X | X | X | X |  |
| Co-Immunoprecipitation (QMI) | X |  |  | X |  |  | X |  |  | X |  |  |
| Patch-clamping | X |  |  | X |  |  | X | X |  | X |  |  |
| Multi-electrode Array (MEA) | X |  |  | X | X |  | X |  |  | X | X |  |
| High-Throughput Screening | X |  |  |  |  |  | X |  |  |  |  |  |

CTL = Control; CDD = CDKL5 Deficiency Disorder

**Supplementary Table S2 |** Quantitative proteomic profiles of CDD NPCs, neurons and cortical organoids. TMT reporter ion intensity from each peptide normalized for each channel was summed for each protein. Up-regulated proteins are in green cells, and down-regulated proteins are in red cells.

(Excel file Supplementary Table S2)

**Supplementary Table S3 |** Quantitative phosphoproteomic profiles of CDD NPCs, neurons and cortical organoids. TMT reporter ion intensity from each peptide normalized for each channel. Up-regulated phosphoproteins are in green, and down-regulated are in red.

(Excel file Supplementary Table S3)

**Supplementary Table S4 |** Gene ontology over-represented in CDD neurons differentially expressed proteins (DEPs). Results of gene ontology analysis using GOrilla. Grey: FDR>0.05.

(Excel file Supplementary Table S4)

**Supplementary Table S5 |** Ingenuity pathway analysis in the category of nervous system development and function for CDD neurons and cortical organoids.

(Excel file Table Supplementary S5)

**Supplementary Table S6 |** Gene ontology analysis of significantly regulated phosphoproteins in CDD neurons and cortical organoids. Results of gene ontology analysis using GOrilla. Grey: FDR>0.05.

(Excel file Table Supplementary S6)

**Supplementary Table S7 |** Neuronal signaling compound library (SelleckChem).

(Excel file Table Supplementary S7)

**Supplementary Table S8 |** Drug ontology of lead compounds.

(Excel file Table Supplementary S8)

**Supplementary Table S9 |** Antibodies used in this study.

| **Antibody** | **Source** | **Catalog Number** |
| --- | --- | --- |
| Rabbit anti-Lin28 | Abcam | ab46020 |
| Goat anti-Nanog | R&D Systems | aF1997 |
| Mouse anti-Nestin | Abcam | ab22035 |
| Rabbit anti-Sox2 | Cell Signalling | 2748 |
| Rabbit anti-GFAP | DAKO | Z033429 |
| Chicken anti-MAP2 | Abcam | ab5392 |
| Rat anti-CTIP2 | Abcam | ab18465 |
| Rabbit anti-S6 Ribosomal Protein (5G10) | Cell Signaling | 2217 |
| Rabbit anti-Phospho-S6 Ribosomal Protein (Ser240/244) | Cell Signaling | 2215 |
| Rabbit anti-p70S6 Kinase Protein | Cell Signaling | 9202 |
| Rabbit anti-Phospho-p70S6 Kinase Protein (Thr421/Ser424) | Cell Signaling | 9204 |
| Rabbit anti-4E-BP1 Protein | Cell Signaling | 9452 |
| Rabbit anti-Phospho-4E-BP1 Protein (Thr37/46) | Cell Signaling | 9459 |
| Mouse anti-actin | Abcam | ab8226 |
| Rabbit anti-Trimethyl-Histone H3 (Lys27) | Millipore | 07-449 |
| Mouse anti-CDKL5 | Santa Cruz | sc-376314 |
| Chicken anti-GFP | Abcam | ab13970 |
| Rabbit anti-Synapsin1 | Millipore | AB1543P |
| Mouse anti-PSD-95 | NeuroMab | 75-028 |
| Mouse anti-Vglut1 | Synaptic Systems | 135311 |
| Rabbit anti-Homer1 | Synaptic Systems | 160003 |
| Donkey anti-Goat IgG-Alexa Fluor 488 | Thermo Fisher | A11055 |
| Donkey anti-Mouse IgG- Alexa Fluor 488 | Thermo Fisher | R37114 |
| Donkey anti-Rabbit IgG- Alexa Fluor 488 | Thermo Fisher | R37118 |
| Donkey anti-Rat IgG- Alexa Fluor 488 | Thermo Fisher | A21208 |
| Goat anti-Mouse IgM- Alexa Fluor 488 | Thermo Fisher | A21042 |
| Goat anti-Chicken IgY- Alexa Fluor 488 | Thermo Fisher | A11039 |
| Donkey anti-Rabbit IgG- Alexa Fluor 555 | Thermo Fisher | A31572 |
| Donkey anti-Mouse IgG- Alexa Fluor 555 | Thermo Fisher | A31570 |
| Donkey anti-Mouse IgG- Alexa Fluor 647 | Thermo Fisher | A31571 |
| Donkey anti-Rabbit IgG- Alexa Fluor 647 | Thermo Fisher | A31573 |
| Goat anti-Mouse IgM- Alexa Fluor 647 | Thermo Fisher | A21238 |
| Goat anti-Chicken IgY- Alexa Fluor 647 | Thermo Fisher | A21449 |
