# Supplementary figures and images for: Altered network and rescue of human neurons derived from individuals with early-onset genetic epilepsy
Source: Mol Psychiatry. 2021 Apr 22;26(11):7047–68. doi: 10.1038/s41380-021-01104-2 (PMC8531162; doi:10.1038/s41380-021-01104-2)

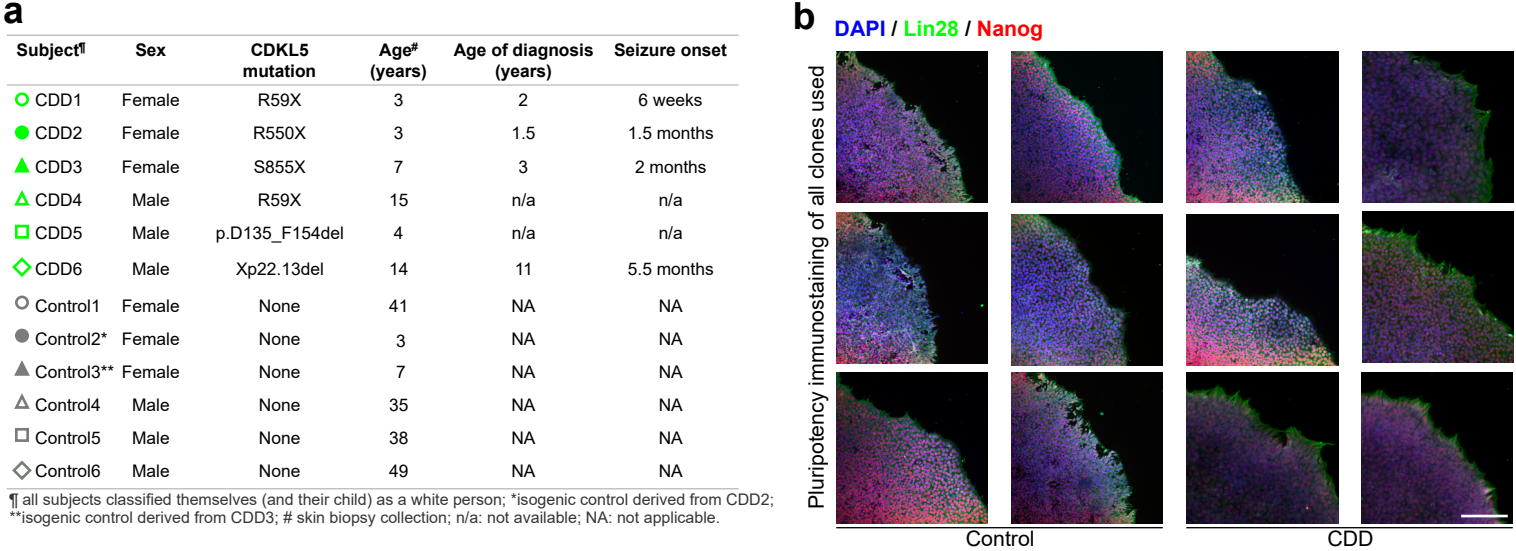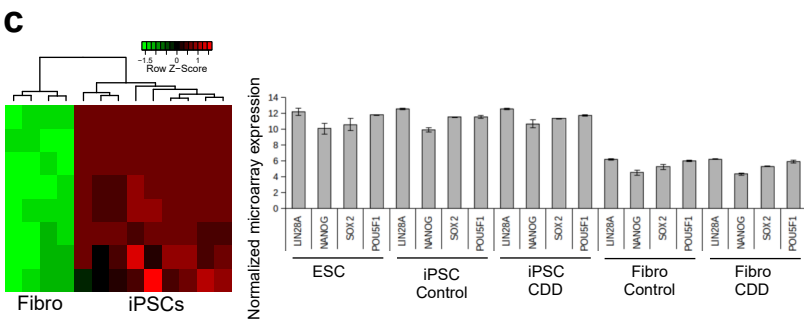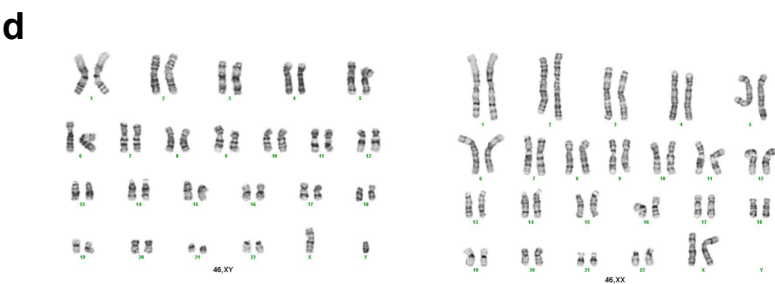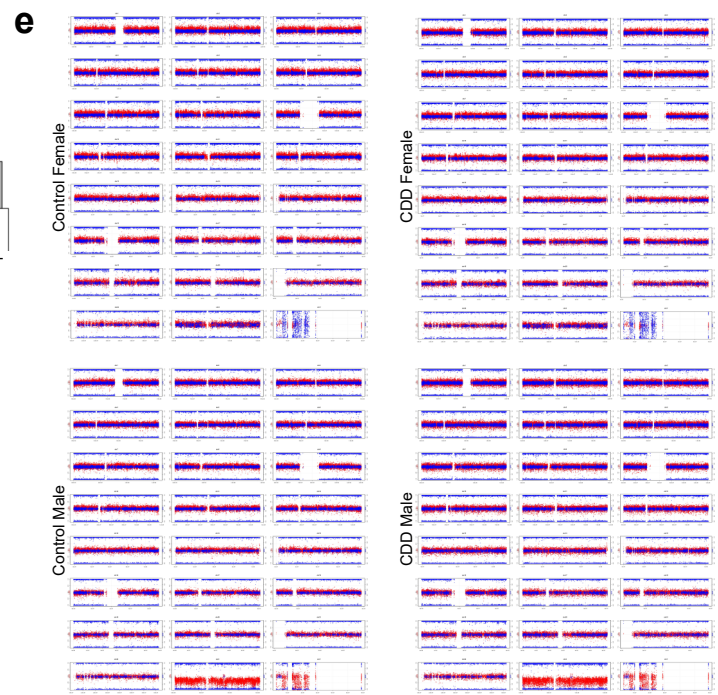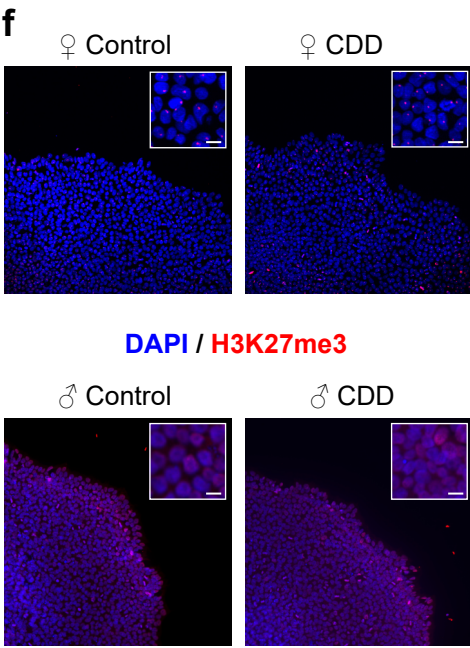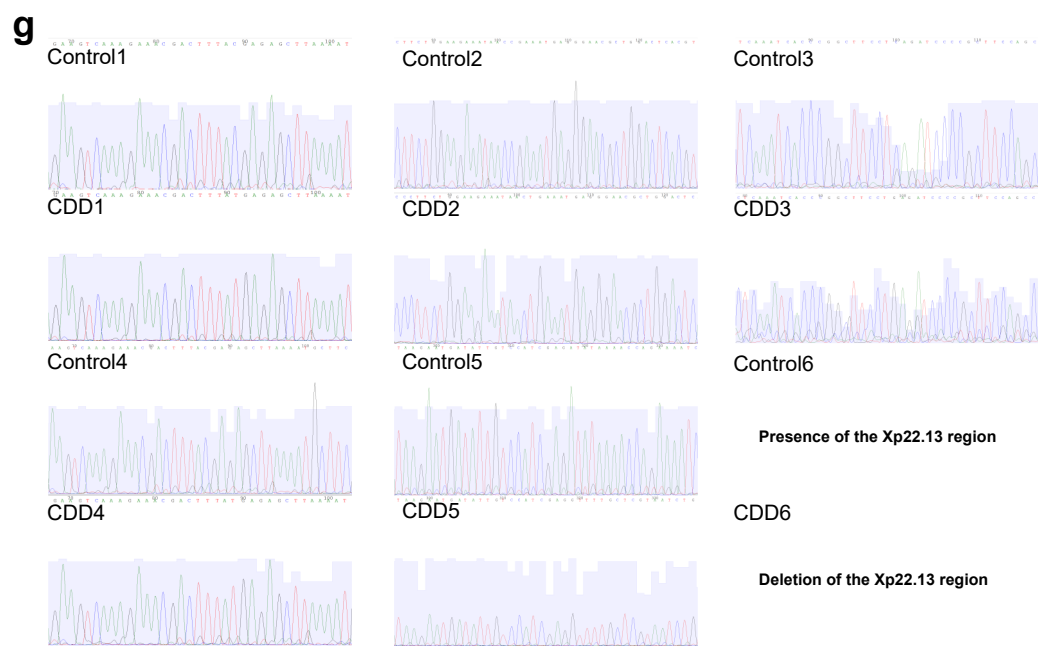

Supplement: Supplementary file 2 — Supplemental figure 1 [file 41380_2021_1104_MOESM2_ESM.pdf]

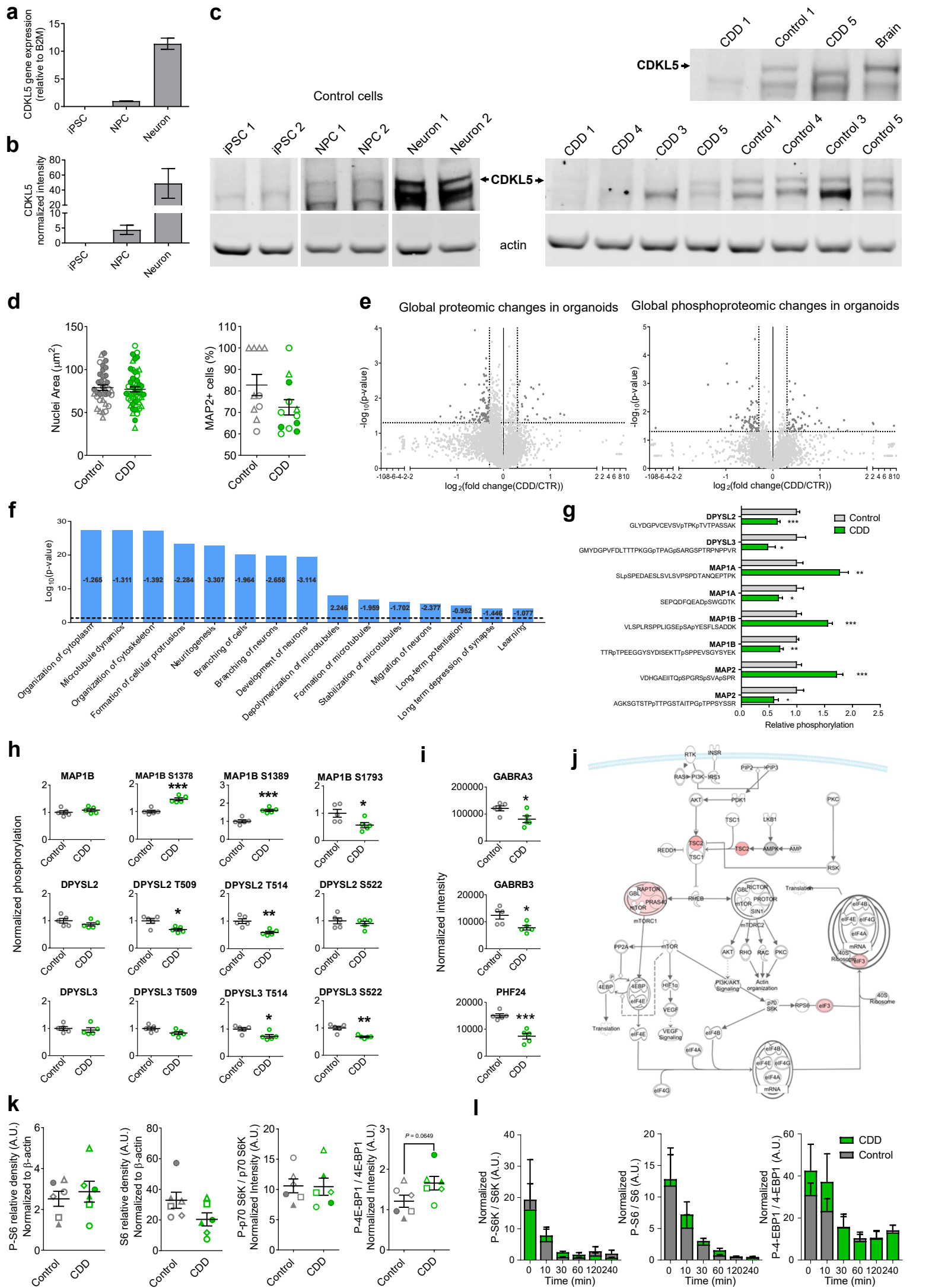

Supplement: Supplementary file 3 — supplemental figure 2 [file 41380_2021_1104_MOESM3_ESM.pdf]

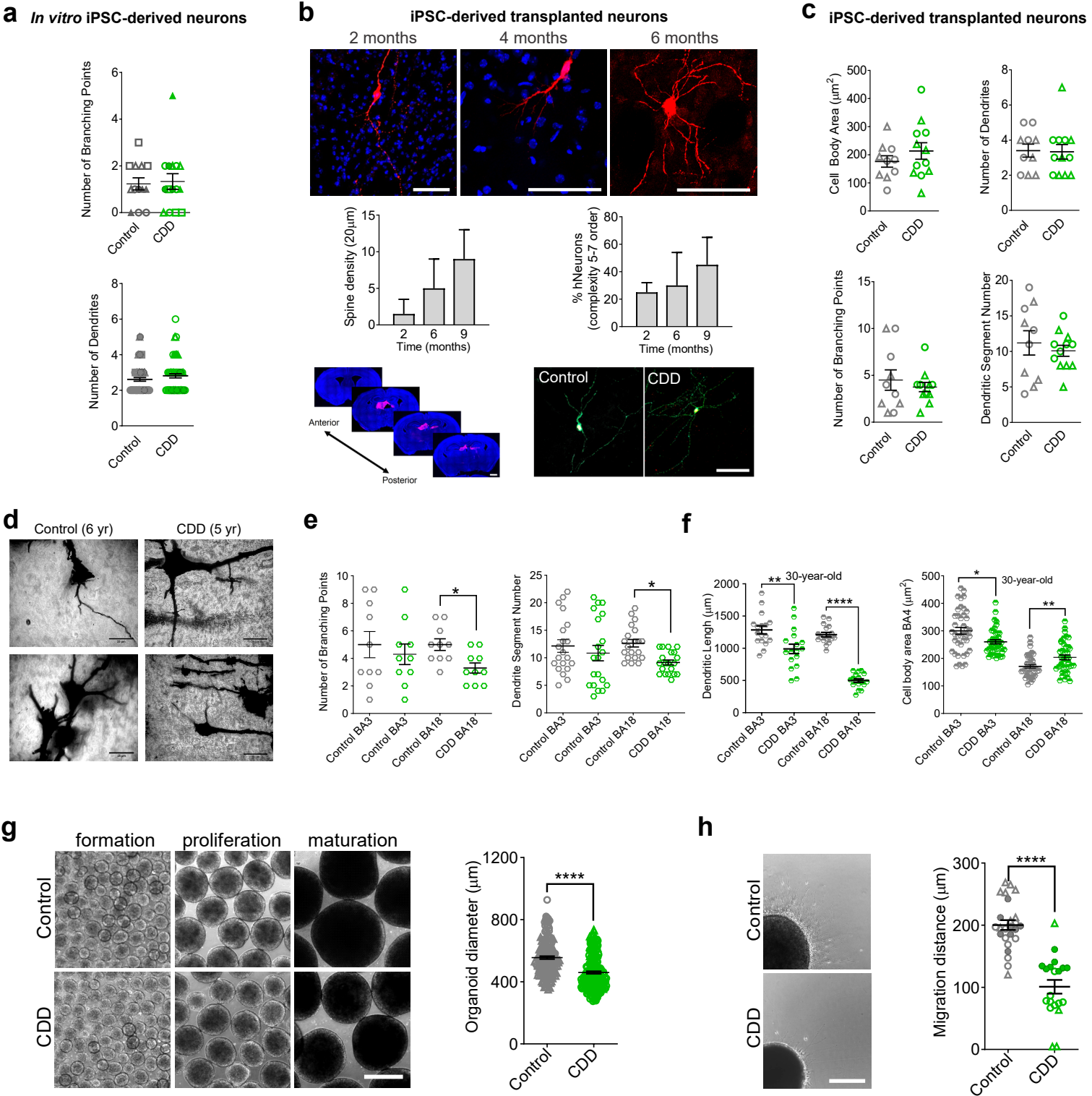

Supplement: Supplementary file 4 — Supplemental Figure 3 [file 41380_2021_1104_MOESM4_ESM.pdf]

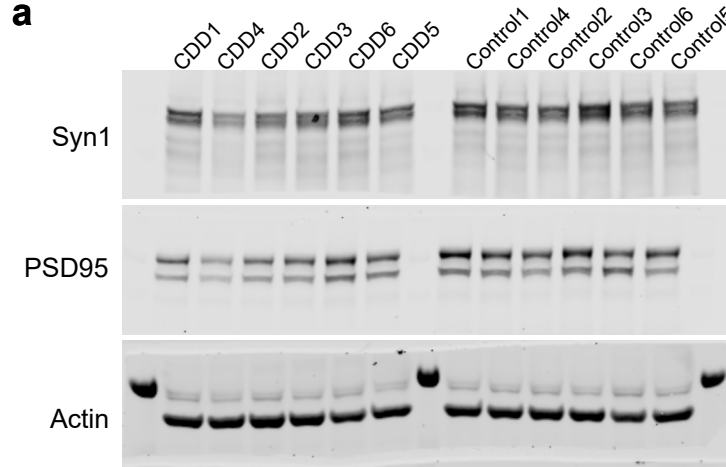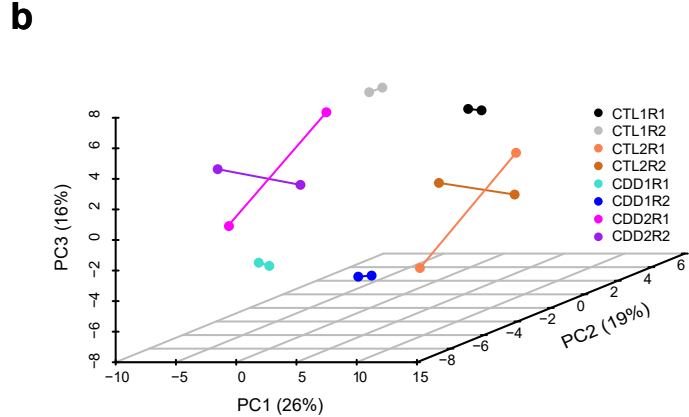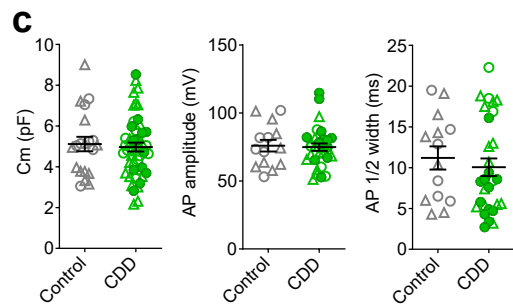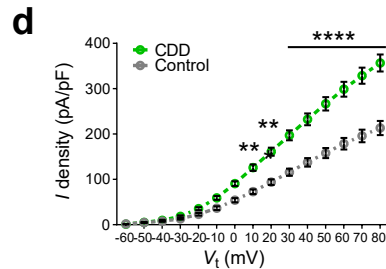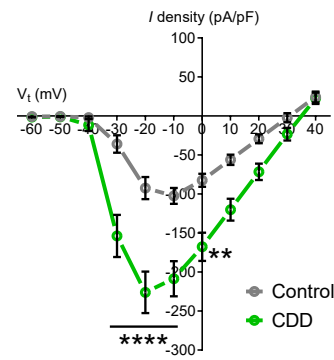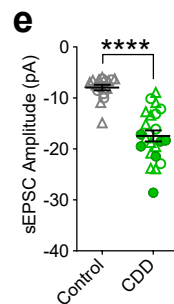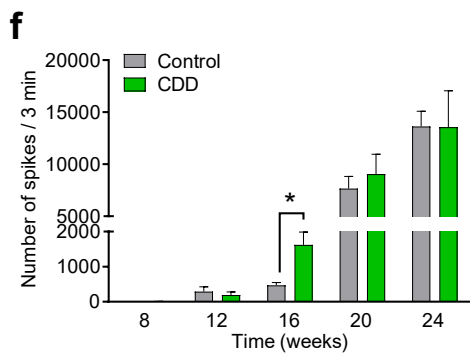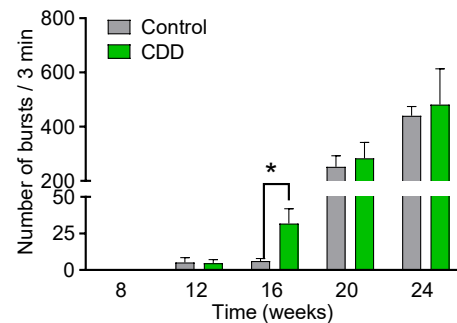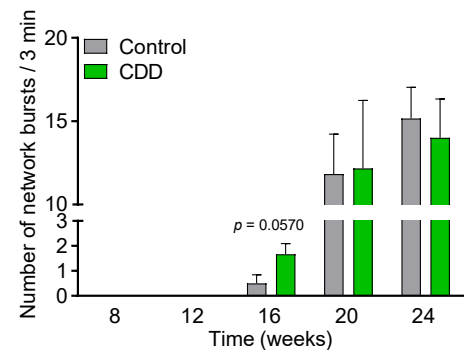

Supplement: Supplementary file 5 — Supplemental Figure 4 [file 41380_2021_1104_MOESM5_ESM.pdf]

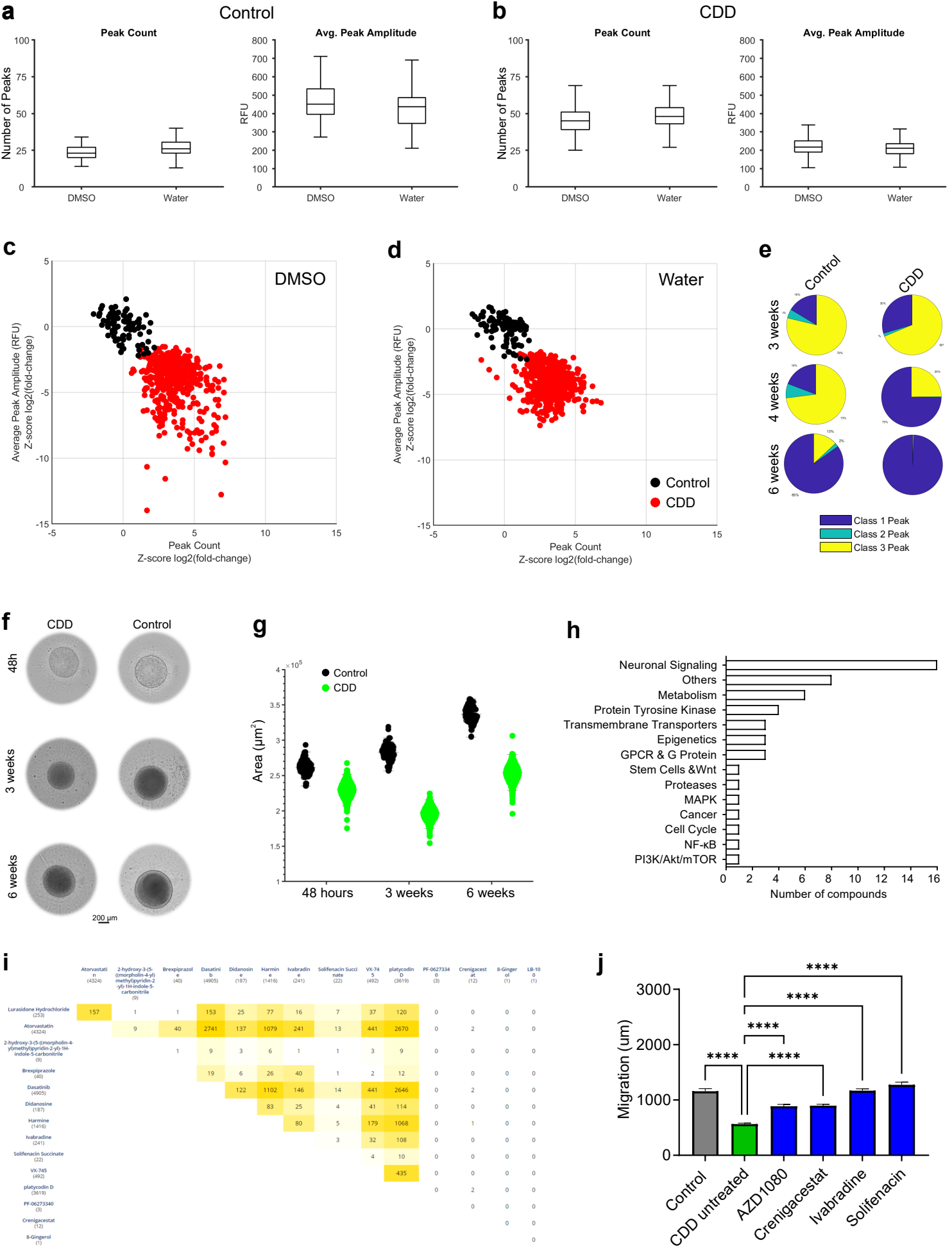

Supplement: Supplementary file 6 — Supplemental Figure 5 [file 41380_2021_1104_MOESM6_ESM.pdf]
